# Identifying new biomarkers of aggressive Group 3 and SHH medulloblastoma using 3D hydrogel models, single cell RNA sequencing and 3D OrbiSIMS imaging

**DOI:** 10.1186/s40478-022-01496-4

**Published:** 2023-01-11

**Authors:** Franziska Linke, James E. C. Johnson, Stefanie Kern, Christopher D. Bennett, Anbarasu Lourdusamy, Daniel Lea, Steven C. Clifford, Catherine L. R. Merry, Snow Stolnik, Morgan R. Alexander, Andrew C. Peet, David J. Scurr, Rian L. Griffiths, Anna M. Grabowska, Ian D. Kerr, Beth Coyle

**Affiliations:** 1 Children’s Brain Tumour Research Centre, School of Medicine, Biodiscovery Institute, University of Nottingham, Nottingham, UK; 2 School of Pharmacy, University of Nottingham, Nottingham, UK; 3 Institute of Cancer and Genomic Sciences, University of Birmingham, Birmingham, UK; 4 Birmingham Children’s Hospital, Birmingham, UK; 5 Digital Research Service, University of Nottingham, Nottingham, UK; 6 Wolfson Childhood Cancer Research Centre, Translational & Clinical Research Institute, Newcastle University Centre for Cancer, Newcastle Upon Tyne, NE1 7RU UK; 7 School of Medicine, Biodiscovery Institute, University of Nottingham, Nottingham, UK; 8 School of Life Sciences, University of Nottingham, Nottingham, UK

**Keywords:** Medulloblastoma, Group 3, SHH, Tricarboxylic acid (TCA) cycle, Small leucine-rich proteoglycans (SLRPs)

## Abstract

The most common malignant brain tumour in children, medulloblastoma (MB), is subdivided into four clinically relevant molecular subgroups, although targeted therapy options informed by understanding of different cellular features are lacking. Here, by comparing the most aggressive subgroup (Group 3) with the intermediate (SHH) subgroup, we identify crucial differences in tumour heterogeneity, including unique metabolism-driven subpopulations in Group 3 and matrix-producing subpopulations in SHH. To analyse tumour heterogeneity, we profiled individual tumour nodules at the cellular level in 3D MB hydrogel models, which recapitulate subgroup specific phenotypes, by single cell RNA sequencing (scRNAseq) and 3D OrbiTrap Secondary Ion Mass Spectrometry (3D OrbiSIMS) imaging. In addition to identifying known metabolites characteristic of MB, we observed intra- and internodular heterogeneity and identified subgroup-specific tumour subpopulations. We showed that extracellular matrix factors and adhesion pathways defined unique SHH subpopulations, and made up a distinct shell-like structure of sulphur-containing species, comprising a combination of small leucine-rich proteoglycans (SLRPs) including the collagen organiser lumican. In contrast, the Group 3 tumour model was characterized by multiple subpopulations with greatly enhanced oxidative phosphorylation and tricarboxylic acid (TCA) cycle activity. Extensive TCA cycle metabolite measurements revealed very high levels of succinate and fumarate with malate levels almost undetectable particularly in Group 3 tumour models. In patients, high fumarate levels (NMR spectroscopy) alongside activated stress response pathways and high Nuclear Factor Erythroid 2-Related Factor 2 (NRF2; gene expression analyses) were associated with poorer survival. Based on these findings we predicted and confirmed that NRF2 inhibition increased sensitivity to vincristine in a long-term 3D drug treatment assay of Group 3 MB. Thus, by combining scRNAseq and 3D OrbiSIMS in a relevant model system we were able to define MB subgroup heterogeneity at the single cell level and elucidate new druggable biomarkers for aggressive Group 3 and low-risk SHH MB.

**Supplementary Information:**

The online version contains supplementary material available at 10.1186/s40478-022-01496-4.

## Introduction

The most common malignant brain tumour in children, medulloblastoma (MB), is subdivided into 4 subgroups which differ in metastasis patterns and related prognoses [1–3]. Despite these significant subgroup differences, therapy options are still mainly non-targeted cytotoxins and novel approaches are urgently required [4]. Group 3 MB is frequently associated with metastases and has the worst outcome of all MB subgroups with metastasis being an independent predictor of survival in this group of patients [5–7]. Recently, we have shown that MB cell lines grown in 3D hydrogel models mimicking the human brain extracellular matrix can be used to recapitulate the laminar metastatic phenotype of Group 3 MB patients as well as the exclusively nodular phenotype of sonic hedgehog (SHH) MB patients observed in the clinic [8, 9]. Whilst Group 3 tumours are universally high risk, SHH tumours are highly heterogeneous in their outcomes; hence, an ability to delineate risk using biomarkers would be beneficial, particularly if these biomarkers are druggable. Importantly, the 3D hydrogel model recapitulated subgroup-specific adhesion, invasion, chemoresistance and extracellular matrix (ECM) remodelling phenotypes, which make it a tool well-suited to exploring subgroup-specific therapy targets.

Tumour heterogeneity is a major obstacle in cancer treatment and is an area of active research in brain tumours [10, 11]. In our 3D hydrogel models, we also observed that laminar invasion and resistance to chemotherapy were features of distinct subpopulations of Group 3 cells rather than a feature of the global cell population [8]. Consequently, we hypothesized that contrasting therapy responses of the different MB subgroups could be due to both subgroup-specific molecular signalling dependencies and heterogeneity in the underlying cell populations. Novel technologies, such as single cell RNA sequencing (scRNAseq) and label free metabolite imaging (3D OrbiSIMS) allow the analysis of cell phenotypes and functions on the single cell level and therefore visualisation of the full spectrum of brain cancer cell states [12]. This in-depth understanding is important since recurrence and metastasis can only be prevented if therapy approaches target all cancer subpopulations.

Here, we use a combination of state-of-the-art scRNAseq and mass spectrometry imaging (3D OrbiSIMS) to compare 3D MB hydrogels of the highly aggressive Group 3 to their less aggressive SHH counterpart. The 3D OrbiSIMS combines the label free imaging attributes of secondary ion mass spectrometry (SIMS) with the high mass-resolving power of an Orbitrap. Uniquely (amongst mass spectrometry imaging technologies) 3D OrbiSIMS offers the opportunity to probe organic molecules, metabolites in this instance, at high spatial resolution (2 μm) and with high mass resolving power and accuracy (within 5 ppm) [13–15]. The combination of the spatial resolution imaging data obtained from the 3D OrbiSIMS with the single cell gene expression subpopulation data, allowed unique characteristics of both Group 3 and SHH medulloblastoma to be identified. In the SHH sub-group we demonstrated the presence of sub-group specific ECM subpopulations and structures that correlate with good overall survival in patient data, representing a potential biomarker for risk stratification in SHH tumours. In Group 3, we identified metabolism-driven subpopulations by scRNAseq and 3D OrbiSIMS analyses where we also observed fumarate accumulation in Group 3 nodules. NMR spectroscopy confirmed an association between fumarate and poorer overall survival in patients, a feature that could be readily exploited by targeted therapy combinations.

## Materials and methods

### Medulloblastoma cell lines and reagents

DAOY cell line was obtained from ATCC (Manassas, USA), ONS-76 from Dr. Annette Künkele (Charité Universitätsmedizin Berlin, Germany), HD-MB03 from Dr Till Milde (DKFZ Heidelberg, Germany) and D458 from Dr. John R. Silber (University of Washington, Seattle, USA). DAOY and D458 were grown in low glucose DMEM (Thermo Fisher) with 10% FBS (HyClone, Thermo Fisher), ONS-76 and HD-MB03 were grown in low glucose RPMI 1640 (Sigma) with 10% FBS (Thermo Fisher). All cells were maintained at 37 °C in a humidified atmosphere containing 5% CO_2_. During the course of this study, all cell lines were confirmed as negative for mycoplasma contamination (MycoAlert, Lonza).

### HA hydrogel preparation and long-term cell culture

Hydrogels for MB 3D culture were prepared as previously described [8]. Briefly, hyaluronan cross-linked hydrogels were prepared according to the manufacturer’s recommendations (Hystem; BioTime Inc) with 1% Hyaluronan and 2% Extralink (PEGDA) in order to achieve a matrix stiffness of around 1.5 kPa and free diffusion of particles less than 75 kDa [16, 17]. For long-term embedding experiments, a pure gel layer (50 µL) was pipetted and equally distributed in a 24-well plate insert (Greiner). A central cell-containing gel layer was then added (DAOY, ONS-76, D458, HD-MB03: 10,000 cells per 150 µL gel). The “gel sandwich” was finished by adding another pure gel layer on top (50 µL). Each gel layer was allowed to set properly before the next layer was added. Finally, each “gel sandwich” was covered with 100 µl of the appropriate cell medium and the lower well was filled with 300 µl of the appropriate cell medium. The cells were allowed to grow over three weeks with cell medium being replaced every 2–3 days. All gels were monitored daily by visual inspection and photomicroscopy.

### Immunohistochemistry of HA hydrogels

HA hydrogels were removed from the well plate and embedded in Histogel (Thermo Fisher) as described by Pinto et al. [18]. The Histogel blocks were incubated on ice for 10 min, fixed in 4% paraformaldehyde and embedded in paraffin. Sections were further processed for immunohistochemistry. Primary antibodies against Ki67 (1:400; CS9449, Cell Signalling), collagen I (1:100, ab7463), collagen VI (1:100, ab45139) were used in combination with a goat Anti-Rabbit IgG H&L secondary antibody (ab214880; all Abcam except for Ki67). The primary antibody against lumican (1:10, AF2846, R and D systems) was used in combination with a donkey Anti-Goat IgG H&L secondary antibody (1:1000, ab6885).

### Measurement of glucose, lactate, glutamate, glutamine, malate and fumarate in hydrogel supernatants

Secreted metabolites were measured using the Glucose-Glo Assay, the Lactate-Glo Assay, the Glutamine/Glutamate-Glo Assay (all Promega) as well as the Fumarate Detection Assay and the Malate Assay Kit (both Abcam) following the manufacturer’s instructions. To avoid additional background signals, the hydrogels were cultured in phenol-red free medium supplemented with dialyzed FBS (A3382001, Thermo Fisher) for 19 days. Hydrogels were washed three times with PBS and fresh medium was added. Supernatant of hydrogels was collected and snap-frozen 6 h, 24 h, 48 h and 72 h after the medium change. For glucose, lactate and glutamine/glutamate detection samples were diluted at least 1:50 for measurement. Samples or sample dilutions, media controls and standard dilutions were prepared and mixed with assay buffer as described by the manufacturers. The resulting luminescence (Glucose-Glo Assay, Lactate-Glo Assay, Glutamine/Glutamate-Glo Assay) or absorbance (Fumarate Detection Assay, Malate Assay Kit) were measured on a FLUOstar Omega plate reader (BMG Labtech Ltd). For quantification, standard titration curves were prepared for glucose, lactate, glutamine, glutamate, fumarate and malate and included on each plate. Medium samples were measured and subtracted from all samples as background.

### scRNA sequencing and data analysis

Four 3-week hydrogel cultures of ONS-76 and HD-MB03 cells (pools of three gels per cell lines) were transferred into gentleMACS C tubes and processed using the Tumour Dissociation kit and the GentleMACS Dissociation programme (all Miltenyi Biotec) according to the manufacturer’s instructions. Dead cells and cell debris were removed from the resulting suspension of single cells using MS columns and the Dead Cell Removal Kit (both Miltenyi Biotec) according to the manufacturer’s instructions.

Library preparation and sequencing were performed by the Deep Sequencing facility of the University of Nottingham. Briefly, single cell 3’ whole transcriptome sequencing libraries were prepared from dissociated cell suspensions using the Chromium Single Cell 3’ Library and Gel Bead Kit v2, the Chromium Single Cell A Chip Kit and the Chromium Multiplex Kit, 96 rxns (10X Genomics, Pleasanton, California, USA; PN-120267, PN-1000009 and PN-120262). Cell counts and viability estimates were obtained using the LUNA-II Automated Cell Counter (Logos Biosystems), Trypan Blue Stain, 0.4% and Luna Cell Counting Slides (Logos Biosystems; T13001 and L12001). Live cell counts were used to calculate cell input, rather than total cell count, as visual inspection of cell field on the LUNA II and the gating histogram, showed that > 95% of cells looked viable and that the cell counter appeared to be counting some extracellular debris as non-viable cells. Five thousand input cells were targeted per sample, with the aim of generating sequencing libraries from ~ 2,500 single cells. All steps, including GEM Generation and Barcoding, Post GEM-RT Cleanup and cDNA Amplification and Library Construction were performed according to the Chromium Single Cell 3’ Reagents Kits v2 User Guide, Rev D. Variable steps of this protocol included using 12 cycles of cDNA amplification and 10 cycles of library amplification. Amplified cDNA was quantified using the Qubit Fluorometer and the Qubit dsDNA HS Assay Kit (ThermoFisher Scientific; Q32854) and fragment length profiles were assessed using the Agilent 4200 TapeStation and Agilent High Sensitivity D5000 ScreenTape Assay (Agilent; 5067–5592 and 5067–5593). Completed sequencing libraries were quantified using the Qubit Fluorometer and the Qubit dsDNA HS Assay Kit and fragment length distributions assessed using the Agilent 4200 TapeStation and the High Sensitivity D1000 ScreenTape Assay (Agilent; 5067–5584, 5067–5585).

Libraries were pooled in equimolar amounts and the final library pool was quantified using the KAPA Library Quantification Kit for Illumina Platforms (Roche; KK4824). Libraries were sequenced on the Illumina NextSeq 500 using a NextSeq 500 High Output v2.5 150 cycle kit (Illumina; 20,024,907) to generate > 50,000 raw reads per cell for each sample, using custom sequencing run parameters. Cellranger V3.1.0 (10X Genomics; Genome Reference: hg38; Annotation Source: Ensembl build 94) was used to create fastq files of all cells, to count UMIs for each gene in each cell and create the loupe files. Then Loupe Browser V4.2.0 (10X Genomics) was used to generate t-SNE plots. The Seurat package in R [19] was used to classify cells based on their cell cycle stages. This was performed for both samples following the process outlined by the Satija Lab at (https://satijalab.org/seurat/v3.2/cell_cycle_vignette.html) and as performed in [20].

To determine similarities and differences between cell subpopulations, lists of differentially expressed genes were compared using Multiple List Comparator (MolBioTools) and pairwise intersections were visualized according to the Jaccard index for the nine clusters of ONS-76 (O1-O9) and HD-MB-03 (H1-H9). Since clusters O6 and H5 did not contain significantly differently expressed genes compared to other clusters they were excluded from the further analysis. For each remaining cell cluster pair, the mean Jaccard index was calculated (mean value of Jaccard index for upregulated genes and Jaccard index for downregulated genes). Based on the mean Jaccard value, which indicates similarities in up-and down-regulated genes between two clusters, the number of network connection between these clusters was determined (mean Jaccard > 0.3: 4 connections; mean Jaccard > 0.2: 3 connections; mean Jaccard > 0.15: 2 connections; mean Jaccard > 0.1: 1 connection). The resulting network matrix was visualized in R.

### Cell viability assay and Vincristine and ML385 drug treatment

Three-week old gels were treated with either 10 nM vincristine (VCR) (S1241; Selleckchem), 5 µM NRF2 inhibitor ML385 (6243, Bio-Techne) or both for one week. DMSO was used as a vehicle control. Drugs were renewed 24, 72 and 144 h after the first dose. In order to assess the cell’s recovery potential all gels were washed and covered with fresh, drug-free medium one week after the final VCR treatment and monitored for a further four weeks (total experiment time: 8 weeks). For cell viability assays, Prestoblue reagent (Thermo Fisher) was used according to the manufacturer’s instructions and fluorescence intensity was measured after 40 min incubation time using a microplate reader (FLOUstar Omega; BMG Labtech, Ortenberg, Germany). All gels were washed four times with HBSS buffer (Thermo Fisher) before fresh medium was added to each well. Cell viability within each gel was measured weekly after drug or vehicle treatment.

### Analysis of publicly available data bases

Analysis of available microarray mRNA expression and survival data was conducted using the “R2: Genomics Analysis and Visualization Platform” (http://r2.amc.nl). The Cavalli Medulloblastoma data set was analysed [21]. In this study 763 fresh frozen primary MB samples were hybridised with the Affymetrix Human Gene 1.1 ST Array. For the comparison between MB samples and normal cerebellum the R2: MegaSampler tool (Pfister data set [22] compared to the 9 cerebellum samples from the Roth353 dataset [23]) was used.

### 3D OrbiSIMS imaging

Three-week hydrogel cultures of ONS-76 and HD-MB03 cells were frozen in liquid nitrogen, sectioned (30 µm thick at − 20 °C) and arranged on indium tin oxide coated glass slides. Samples were freeze dried overnight before introduction to the mass spectrometer. 3D OrbiSIMS analysis was performed on a Hybrid SIMS ToF and OrbiTrap instrument (IONTOF GmbH). Calibration of the Orbitrap analyser was performed on a silver sample plate, using silver clusters following the method described by Passarelli et al*.* [13] The Bi_3_^+^ liquid metal ion gun in spectrometry mode and the ThermoFisher Tune software were employed for calibration. For the acquisition of 3D OrbiSIMS images, a 20 keV Ar_3000_^+^ analysis beam was used as primary ion source. Samples were analysed at room temperature across a 200 × 200 µm area in positive and negative polarity. Ar_3000_^+^ primary ions were used with a target current of 0.2 nA with charge compensation performed using a low energy (20 eV) electron flood gun. Duty cycle was set to 38.5% and cycle time to 200 µs. Argon gas flooding was in operation to aid with charge compensation, which led to a pressure of 9.0 × 10^−7^ bar in the main chamber. Optimal target potential varied for different samples. Images were acquired from selected samples at a beam size of either 2 µm or 20 µm diameter, pixel size of 2 µm and each measurement lasted one scan. Mass spectra were recorded at a resolution of 240 000 at m/z 200 in the mass range of 50–750 m/z. The AGC target was off with the maximum injection time set at 500 ms. Assignments were determined by accurate mass within 3 ppm error of the calculated mass. Both data acquisition and the subsequent data processing were performed using SurfaceLab 7 software (IONTOF GmbH). Orbitrap data was acquired using a Thermo Fisher Orbitrap HF mass spectrometer. Visualisation of the nodules was assisted by microscopy (Olympus, CKX41 with attached Canon camera, DS126431).

### HR-MAS NMR spectroscopy of fumarate levels in MB patients

Frozen diagnostic tumour tissue for 60 medulloblastoma cases were acquired from the CCLG tissue bank (CCLG 2015 BS 05). Tissue was snap frozen in liquid nitrogen shortly after surgical resection and stored at − 80 °C. Tissue had been removed prior to the patient receiving any chemo- or radiotherapy.

HR-MAS was performed at the Henry Wellcome Building Biomolecular NMR Facility at the University of Birmingham. Tissue was cut with a scalpel over dry ice to fit into a 12 μl or 50 μl zirconium rotor before being weighed. The internal standard 3-(Trimethylsilyl)propionic-2,2,3,3-d4 acid sodium salt (TMSP) (Cambridge Biosciences, Cambridge, UK) was added to the sample in a rotor dependent manner. 3 μl of standard was added to 12 μl rotors whilst 5 μl of standard was added to 50 μl rotors. D_2_O (Sigma Aldrich, Dorset, UK) was added to completely fill the rotor before it was fully assembled. The sample and rotor were kept cold over dry ice during preparation to prevent tissue degradation.

Spectra were acquired using a Bruker Avance spectrometer (Bruker, Coventry, UK) with a magnetic field strength of 500 MHz fitted with a 4 mm three channel HCD HRMAS z-PFG band probe. The rotor was spun at a temperature of 4 °C to prevent metabolic activity and a frequency of 4800 Hz to remove spinning sidebands from the spectra. A NOESY pulse sequence was used with 2 s pre-saturation to suppress the water signal and a repetition time of 4 s. 256 or 512 averages were acquired for 50 μl and 12 μl rotors respectively. Using this protocol, an experiment with 256 averages was completed in 17 min.

Free Induction Decays were Fourier transformed in Topsin 2.0 (Bruker, Coventry, UK) and the resulting spectra were imported into MestReNova 9.0.1 software suite (Mestrelab Research, Spain). To ensure quality control, the spectra were visually examined for a high signal to noise ratio, a well-defined TMSP peak and clear discrimination of the choline, phosphocholine and glycerophosphocholine peaks at 3.2–3.3 ppm. The spectra were phased, baseline corrected and the chemical shift referenced with respect to TMSP at 0 ppm. Fumarate was fit as a singlet peak at 6.5 ppm, and quantified by comparing the area of the fumarate peak to the area of the internal standard peak, taking into account the number of protons contributing to both fumarate and the standard signal.

Fumarate concentration was normalised by dividing by sample mass. The normalised fumarate concentration was compared between medulloblastoma subgroups using a non-parametric Kruskal–Wallis test. The cohort was stratified into samples with no quantifiable fumarate and those with detectable fumarate and subjected to Kaplan–Meier tests for the whole cohort and each subgroup. Hazard Ratios for the two groups were calculated using Cox Proportional Hazards models. Fishers Exact Tests were performed to test for associations between the presence of detectable fumarate and metastatic disease, large cell anaplastic histology and MYC oncogene amplification.

### Generation of diagrams

The diagram in Fig. 4d was generated using the online software BioRender® (https://biorender.com/).

### Statistics

Data were expressed as means ± SEM. The biological number of samples and corresponding statistical test and significance level is indicated in each figure legend. All statistical analyses and plots were carried out with GraphPad Prism 9.00 (GraphPad Software Inc., La Jolla, CA, USA) unless stated otherwise. Kaplan Meier survival data analysis was performed using the R2: Genomics Analysis and Visualization Platform (http://r2.amc.nl) and statistical significance was tested using the logrank test as described in Bewick et al. [24].

## Results

### scRNAseq analysis of medulloblastoma models reveals independent, metabolic subpopulations in Group 3 and SHH-specific extracellular matrix (ECM) subpopulations

**Fig. 1:**
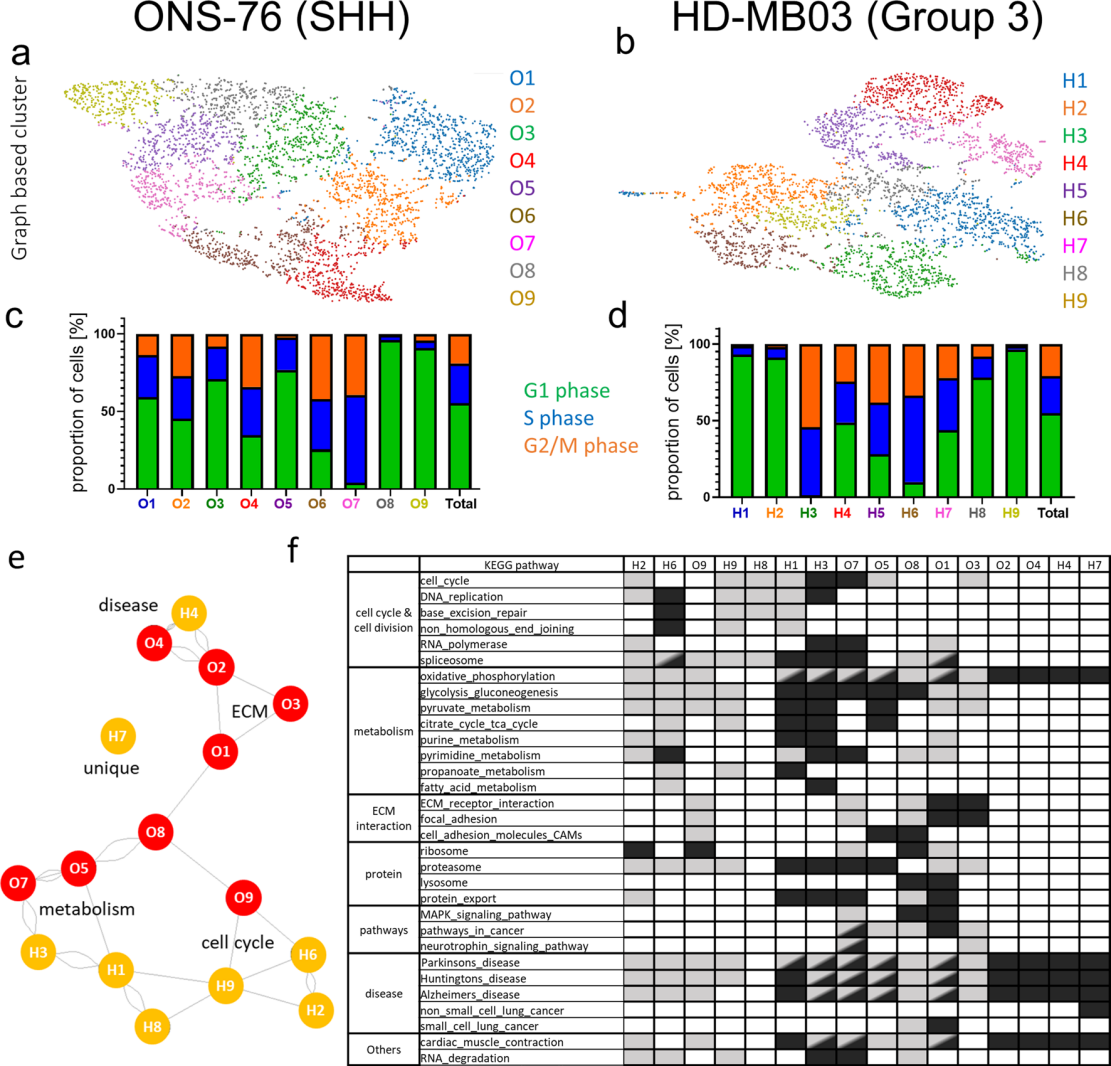
scRNA sequencing reveals tumour subpopulations characterized by specific gene expression patterns in SHH and Group 3 MB models. Analysis of scRNA sequencing data from ONS-76 (SHH) cells **a** and HD-MB03 (Group 3) cells **b** growing as 3D hydrogel models identified 9 different graph-based clusters for each cell line (t-SNE plot, see also Additional file 1: Table S1 for cluster characteristics). Gene expression of each cell was used to annotate a particular cell cycle phase. Relative proportion of cells in G1, S and G2/M phase for ONS-76 **c** and HD-MB03 **d** cells highlights heterogeneity between clusters (see Additional file 2: Fig. S1 for t-SNE plot of cell cycle annotation). **e** The network graph highlights similar clusters based on their gene expression (Intersection Tables with Jaccard Indices used to create the network graph are shown in Additional file 2: Fig. S2). Note that clusters O4 and H4 are most similar while cluster H7 is mostly unique in its gene expression compared to all other clusters. (The network graph shows cluster similarity based on the Jaccard indices of shared up-and downregulated genes with the number of connections indicating higher Jaccard values [1: 0.15 < Jaccard ≥ 0.1; 2: 0.2 < Jaccard ≥ 0.15; 3: 0.3 < Jaccard ≥ 0.2; 4: Jaccard ≥ 0.3]). **f** Extract of Top20 KEGG pathways of significantly up-(dark grey) and down-(light grey) regulated genes are listed for each cluster (full overview shown in Additional file 2: Fig. S3). Note the unique presence of ECM and adhesion subpopulations in ONS-76 clusters while metabolic clusters are dominant in HD-MB03

In our study we used validated medulloblastoma cell lines representing the SHH and Group 3 medulloblastoma subgroups [8]. Three weeks after initiation, hydrogel models of ONS-76 (SHH) and HD-MB03 (Group 3) cells displayed heterogeneous growth and migration; to explore this behaviour at the gene expression level these models were then analysed using scRNAseq. We identified 9 different cell clusters in each model based on gene expression similarities and determined significantly up- and downregulated genes between the clusters (Additional file 1: Table S1, Fig. S1 a–b). Importantly, the SHH and Group 3 hydrogel models express different levels of subgroup-specific gene markers for the SHH pathway (*SMO, PTCH1, SFRP2*) and Group 3 (*MYC, OTX2, GNB3*) which is in line with previously performed bulk RNA sequencing data [8] (Additional file 2: Fig. S1 a–f; Additional file Methods). Based on gene expression each cell was assigned to a cell cycle phase and the cell cycle phase distribution was compared between clusters (Fig. 1 c–d, Additional file 2: Fig. S1g). Interestingly, both hydrogel models contained clusters with similar and very specific cell cycle distributions, such as clusters O7 and H3 that contained very few cells in G1. To identify general cluster similarities, all significantly up-and downregulated genes were compared between clusters, and clusters with shared up- or downregulated genes were connected in a network graph (Additional file 2: Fig. S2 a–f). In the SHH model all clusters are connected with at least one other cluster and clusters O5 and O7 display highest similarities (Additional file 2: Fig. S2c). In contrast, two Group 3 clusters, H4 and H7, are very distinct from other Group 3 clusters while cluster H9 shares similarities with 4 other clusters and could represent an intermediate state (Additional file 2: Fig. S2f). Interestingly, the comparison of cell clusters across models shows strong concordance between clusters O4, O2 and H4 as well as between O7 and H3 as was also predicted from the cell cycle analysis (Fig. 1c–e, Additional file 2: Fig. S2g–h). Notably, any similarities and differences observed between clusters were independent of their cell cycle status. KEGG pathway analysis of cluster-specific gene lists confirms some shared functional pathways between SHH and Group 3 MB models but also highlights subgroup-specific cellular functions (Fig. 1f, Additional file 2: Fig. S3). Some clusters share KEGG pathway similarities with neurological diseases such as Alzheimer’s disease (O4, O2, H4) or metabolism (H1, H3 and to a lesser degree O5, O7). Importantly, clusters H1 and H3 are the strongest metabolic clusters with the most gene expression changes related to metabolism and no comparable counterpart identified in the ONS-76 (SHH) model. Conversely, whilst enhanced metabolic clusters seem to be characteristic of the Group 3 model, clusters characterized by ECM components and adhesion pathways are exclusively found in the SHH model (O1, O3, O8; Fig. 1e–f).

Taken together, the scRNAseq data show that although SHH and Group 3 models harbour some comparable subpopulations with shared functions, these exist alongside subgroup-specific clusters indicating heterogeneity in metabolism genes (Group 3 models) and tumour microenvironment interactions (SHH models).

### Subgroup-specific features of the tumour composition are identified by metabolic imaging of 3D medulloblastoma hydrogel models

Although scRNAseq data delivers a great level of in-depth information on distinct cell clusters, their spatial location within the hydrogel is lost. In order to obtain metabolite distribution within each model the hydrogel samples were analysed by 3D OrbiSIMS to obtain mass spectrometry imaging (MSI) data. Three weeks after initiation, hydrogel models of ONS-76 (SHH) and HD-MB03 (Group 3) (Additional file 2: Fig. S4a–b) were snap frozen, sectioned, and analysed under cryogenic conditions [13]. The MSI data acquired enabled the detection of metabolite distribution both within and around the nodules and we were able to successfully resolve ions that exist at a similar m/z value (Additional file 2: Fig. S4c).

**Fig. 2:**
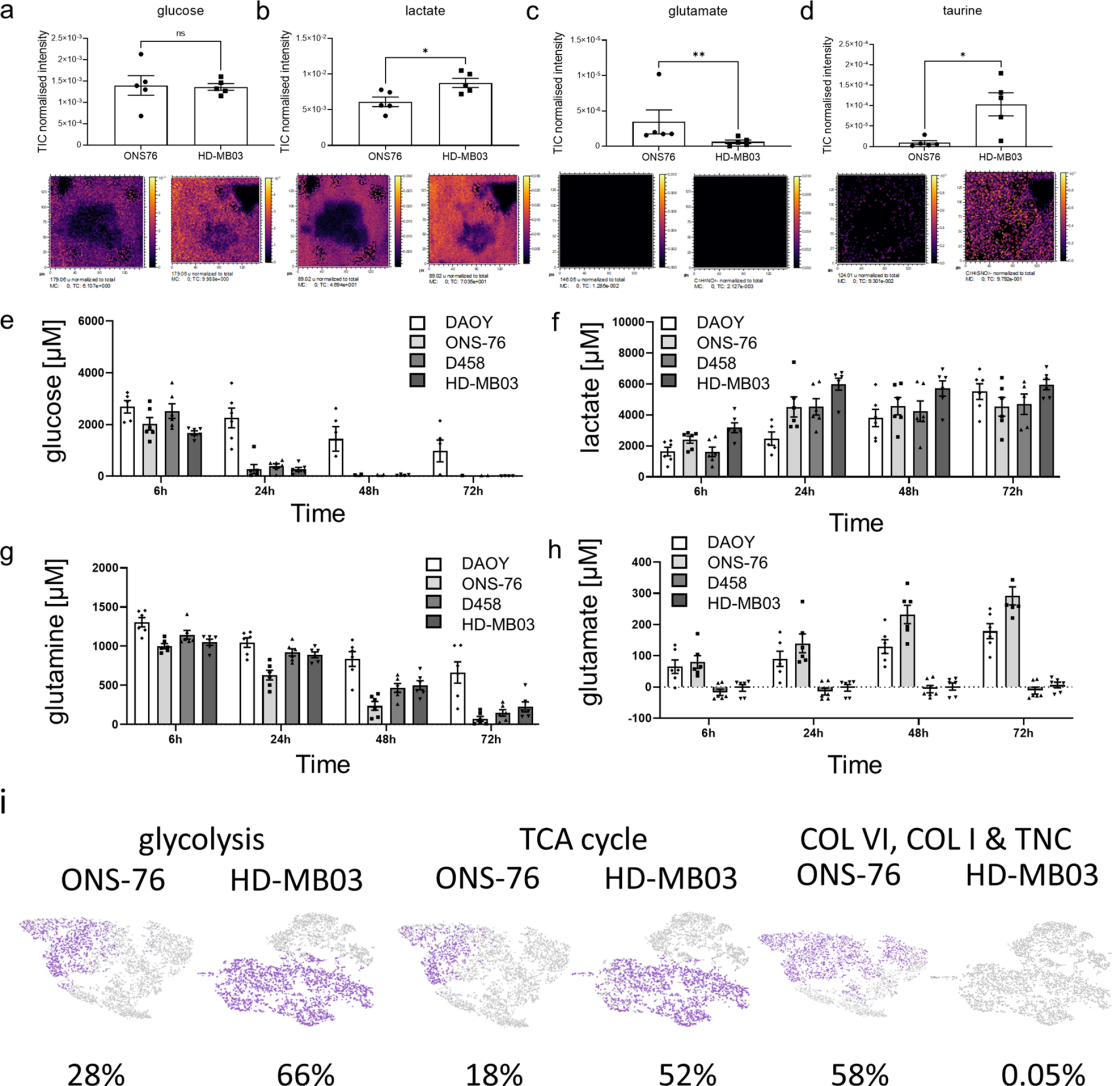
The 3D hydrogel models display subgroup-specific metabolic characteristics to those observed in MB patients. Several metabolites are known to be specifically high in MB patients [25]. 3D OrbiSIMS mass spectrometry imaging also confirms relatively high metabolite levels of glucose **a**. Lactate levels **b** are elevated in Group 3 nodules (D458, HD-MB03), while glutamate **c** is specifically high in SHH (DAOY, ONS-76). The Group 3-specific metabolite taurine **d** is also significantly higher in Group 3 nodules compared to SHH (a–d: mean ± SEM; *n* = 5; a, b: unpaired t-test, c: Mann Whitney test, d: unpaired t-test with Welch correction; *P < 0.05, **P < 0.01). Gel supernatants of 3 week-old SHH and Group 3 nodules were collected 6, 24, 48 and 72 h after medium change (containing 5000 µM glucose and 2000 µM glutamine) to measure consumption and secretion of glucose **e**, lactate **f,** glutamine **g** and glutamate **h**. Note that although both subgroups quickly consume glucose and glutamine, only lactate is secreted by both. Interestingly, glutamate secretion is characteristic for SHH nodules and below detection limit in Group 3 nodules (mean ± SEM; *n* = 3). **i** Gene expression for glycolysis (ALDOA, LDHB, GALM, AKR1A1, HKDC1, ENO2, ALDH2, ENO3, PGAM2, PFKM, PDHA1, PCK2, ALDH9A1), TCA cycle (SUCLG1, PDHA1, PCK2, MDH2, PC, MDH1, FH) and ECM genes (COL6A1, COL6A2, COL6A3, COL1A1, TNC) at the single cell level are displayed for the SHH (ONS 76) and Group 3 (HD MB03) model (purple: gene expressing cell; grey: non-expressing cell; percentage indicates relative proportion of total cells expressing the specified gene set). Cluster specific expression of these gene sets is shown in Additional file 2: Fig. S6

In order to identify further subgroup specific cluster differences, targeted analysis was undertaken of the acquired 3D OrbiSIMS data to specifically identify metabolites that have been previously shown to be differentially expressed in MB patients by MRI analysis [25]. Firstly, the expected high levels of choline, that typify high grade tumours including medulloblastoma [26], were observed within both SHH and Group 3 MB models (Additional file 2: Fig. S5). As observed in Group 3 patients, we also observed high levels of glucose, and significantly higher levels of lactate and taurine in the extracellular matrix surrounding Group 3 models relative to SHH[25] (Fig. 2a–d, Additional file 2: Fig. S5c). Interestingly, higher glutamate levels in SHH patients in comparison to Group 3 patients [25], were also replicated in the extracellular matrix of SHH 3D hydrogel models (Fig. 2c). Taken together, these findings indicate that the 3D hydrogel model, despite its simplicity, recapitulates some of the clinically-relevant metabolic differences between SHH and Group 3 tumours [25, 26] allowing their analysis in vitro. Inclusion of other tumour microenvironmental cell types will be required to more fully recapitulate metabolic programming in tumours.

The next step, therefore, was to examine differences in energy metabolism by quantifying changes in glycolysis and glutaminolysis over time. To do this, supernatants of SHH and Group 3 models were collected over three days to measure glucose consumption and subsequent lactate secretion (Fig. 2e–f) as well as glutamine consumption and glutamate secretion (Fig. 2g–h). Starting glucose levels (5000 µM) reduced to half in all MB models after 6 h and glucose was almost completely consumed after one day (Fig. 2e). In parallel, lactate levels rose in all MB models, reaching a maximal concentration of approximately 5000 µM after 24 h and remaining stable until day three (Fig. 2f). In comparison glutamine (initial level 2000 µM) was consumed more slowly than glucose (halved after 24 h and completely consumed after 72 h in all apart from DAOY nodules, Fig. 2g). Importantly, secreted glutamate levels rose steadily over time only in the SHH models while remaining low or below detection limit in Group 3 models, thus further confirming subgroup differences in metabolic pathways between SHH and Group 3 MB (Fig. 2h).

The upregulation of metabolic pathways involved in glycolysis and the tricarboxylic acid (TCA) cycle was also strongly observed at the RNA level in Group 3 MB (Fig. 2i, Additional file 2: Fig. S6). In addition, many other pathways that can fuel the TCA cycle, such as fatty acid metabolism, are among the overall upregulated KEGG pathways in Group 3 models (Additional file 2: Fig. S6a). At the single cell level, 66% of HD-MB03 (Group 3) cells expressed genes involved in glycolysis in comparison to 28% of ONS-76 (SHH) cells (Fig. 2i, Additional file 2: Fig. S6b). For gene sets involved in the TCA cycle, metabolic differences became even more striking (52% of HD-MB03 cells expressed TCA genes relative to 18% of ONS-76 cells) (Fig. 2i, Additional file 2: Fig. S6c). In summary, there are clear metabolic differences found exclusively in Group 3 models that may represent therapeutic targets while on the other hand SHH tumours are characterized by a specific matrix components that are worthy of further analysis.

### Small leucine rich proteoglycans and collagens are biomarkers of SHH MB

**Fig. 3:**
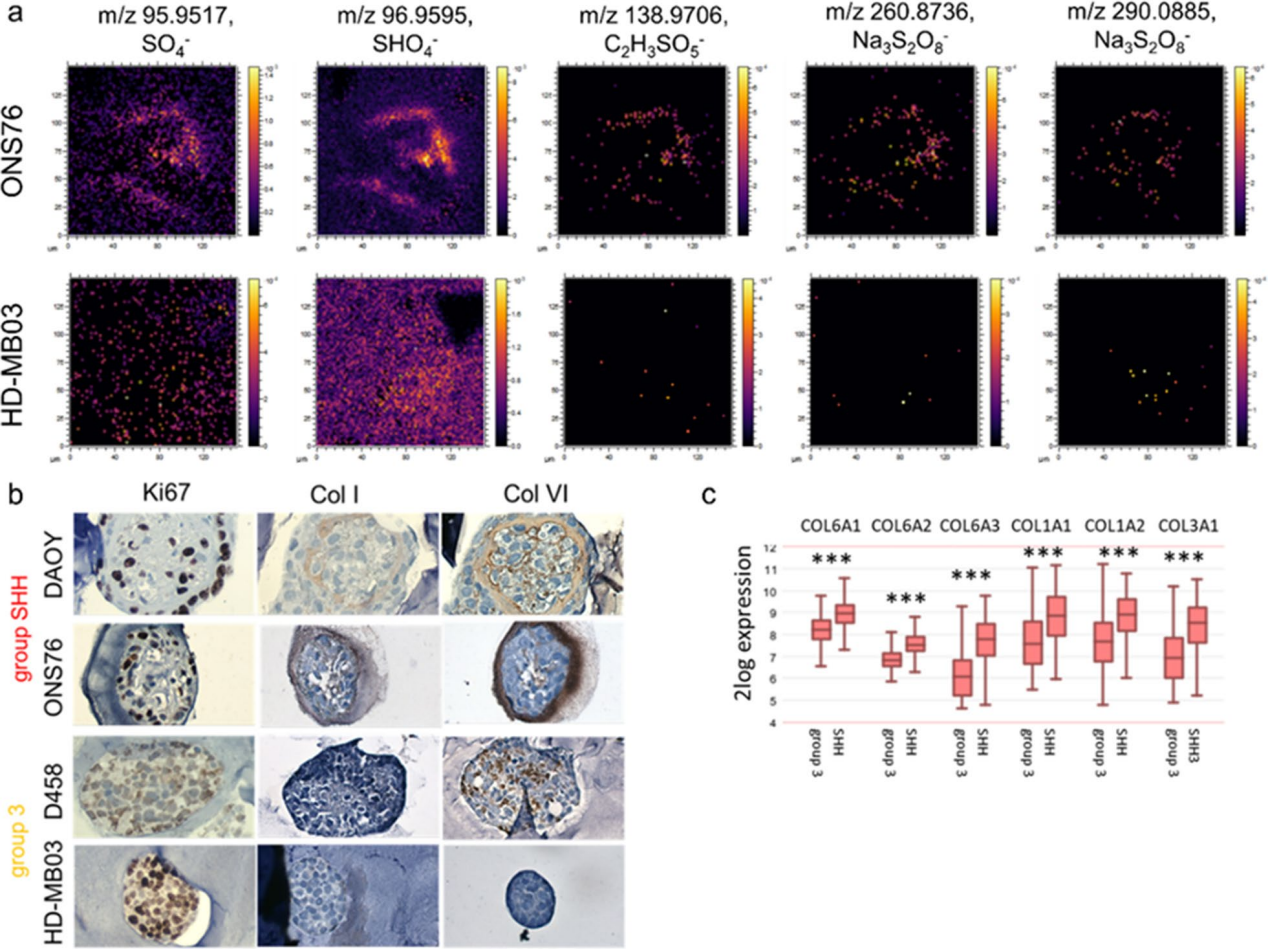
SHH nodules are uniquely characterized by deposits of sulphur-containing species and a collagen-based outer shell. **a** 3D OrbiSIMS mass spectrometry imaging identified several sulphur-containing species within the outer shell of ONS 76 nodules that are non-specifically distributed in HD-MB03 nodules. **b** Similar to the localization of sulphur species, collagens (Col I and Col VI) are specifically located at the outer surface of SHH (DAOY, ONS-76) nodules compared to nonspecific or no expression in Group 3 nodules (D458, HD-MB03). **c** Expression of *COL6A1, COL6A2, COL6A3, COL1A1, COL1A2 and COL3A1* is significantly higher in SHH patients compared to Group 3 patients in the Cavalli data set (Cavalli et al. [21], SHH: *n* = 223 and Group 3: *n* = 144; Kruskal–Wallis test with Dunn’s post hoc test; ***P < 0.001)

**Fig. 4:**
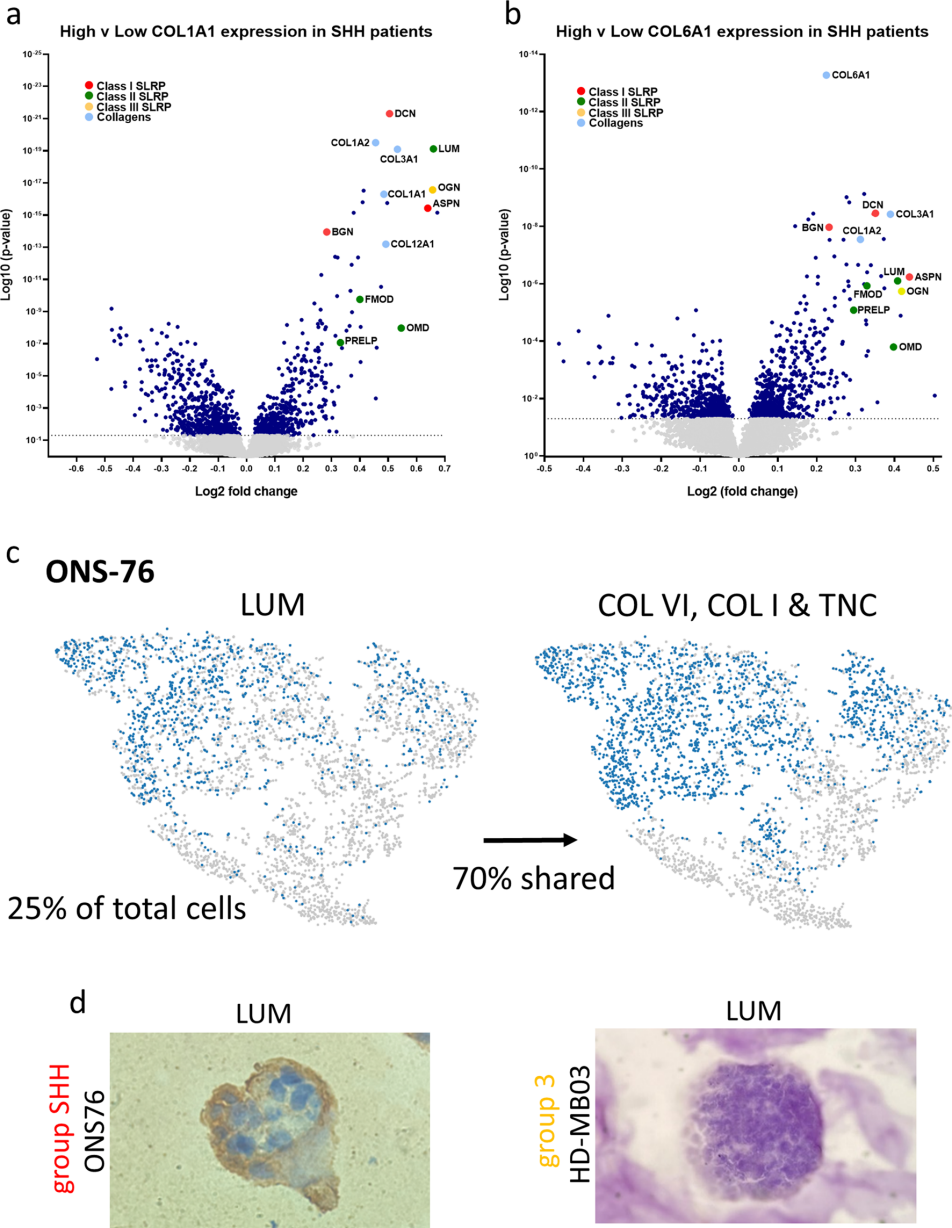
Deposits of sulphur-containing species in SHH nodules are likely to be a combination of small leucine rich proteoglycans including lumican. **a** and **b** Small leucine rich proteoglycans (SLRPs) and other collagens are correlated with high *COL1A1* and *COL6A1* expression in SHH patients in the Cavalli data set (Cavalli et al*.* [21], SHH: *n* = 223, log-rank test used to establish ‘high’ and ‘low’ expression; one-way ANOVA, grey dots represent samples where *p* ≥ 0.05 and navy dots where *p* < 0.05) **c** scRNAseq analysis revealed that *LUM* (a SLRP) is expressed by 25% of cells within SHH nodules and that 70% of these cells also express ECM genes of interest (purple: gene expressing cell; grey: non-expressing cell; percentage indicates relative proportion of total cells expressing the specified gene and percentage ‘shared’ indicates the proportion of *LUM* expressing cells that also express the ECM genes *COL6A1, COL6A2, COL6A3, COL1A1* and *TNC*. **d** Immunohistochemical staining of ONS76 and HD-MB03 hydrogel nodules for lumican reveals that lumican is specifically located on the outer edge of SHH nodules (similar to type-I and type-VI collagens) and is not present in Group 3 nodules

3D OrbiSIMS was also used to identify differences between SHH and Group 3 by unbiased mass analysis, revealing differences in the spatial distribution of several sulphur-containing compounds (Fig. 3a). Sulphur-containing species such as SO_4_^−^ are specifically located at the outer shell of SHH nodules; conversely, these are more diffuse in Group 3 nodules, indicating a different functional role between MB subtypes (Fig. 3a). The location of these sulphur-containing species overlaps with collagens that are also specifically found at the outer shell of SHH nodules, validated by staining (Fig. 3b). Gene expression of these collagens, including type I- and type VI-collagens, is also significantly higher in SHH patients than Group 3 (Fig. 3c, Additional file 2: Fig. S7) and is associated with clusters O1 and O3, which have been identified as SHH-specific ECM receptor interaction and focal adhesion clusters using scRNAseq analysis (Fig. 1e and f). Interestingly, differential gene expression analysis of a genomic patient dataset revealed a strong correlation between the expression of both type I- and type VI-collagens (*COL1A1* and *COL6A1)* and several small leucine rich proteoglycans (SLRPs), including lumican (*LUM*) and fibromodulin (*FMOD*) (Fig. 4a and b), as well as several of the other collagens that are more highly expressed in SHH patients than Group 3 (*COL1A2, COL6A3, and COL3A1)* (Fig. 3c, Fig. s8). Similarly, scRNAseq analysis revealed that within SHH nodules 70% of LUM positive cells co-expressed the genes that defined the ECM cluster (*COL1A1, COL6A1* and *TNC*; Fig. 4c). Lumican and fibromodulin can both contain keratan sulfate (KS) chains and have overlapping but also unique roles in collagen fibrillogenesis, suggesting their KS may be the sulphated component of the shell-like structure that forms around SHH nodules as identified by 3D OrbiSIMS (Fig. 3a) [27–29]. In support of this, immunohistochemical staining of lumican in the ONS-76 and HD-MB03 nodules demonstrated that lumican does indeed form a shell-like structure around SHH nodules, similar to that of type I- and type VI- collagens. In contrast, lumican was absent from Group 3 nodules (Fig. 4d).

Hence, our scRNAseq, 3D OrbiSIMS and IHC data all point to the conclusion that SHH nodules are specifically characterized by a distinct ECM shell-like structure of which type I- and type VI- collagens and SLRPs (lumican) are components.

### Fumarate accumulation during the TCA cycle as a novel target of Group 3 medulloblastoma

**Fig. 5:**
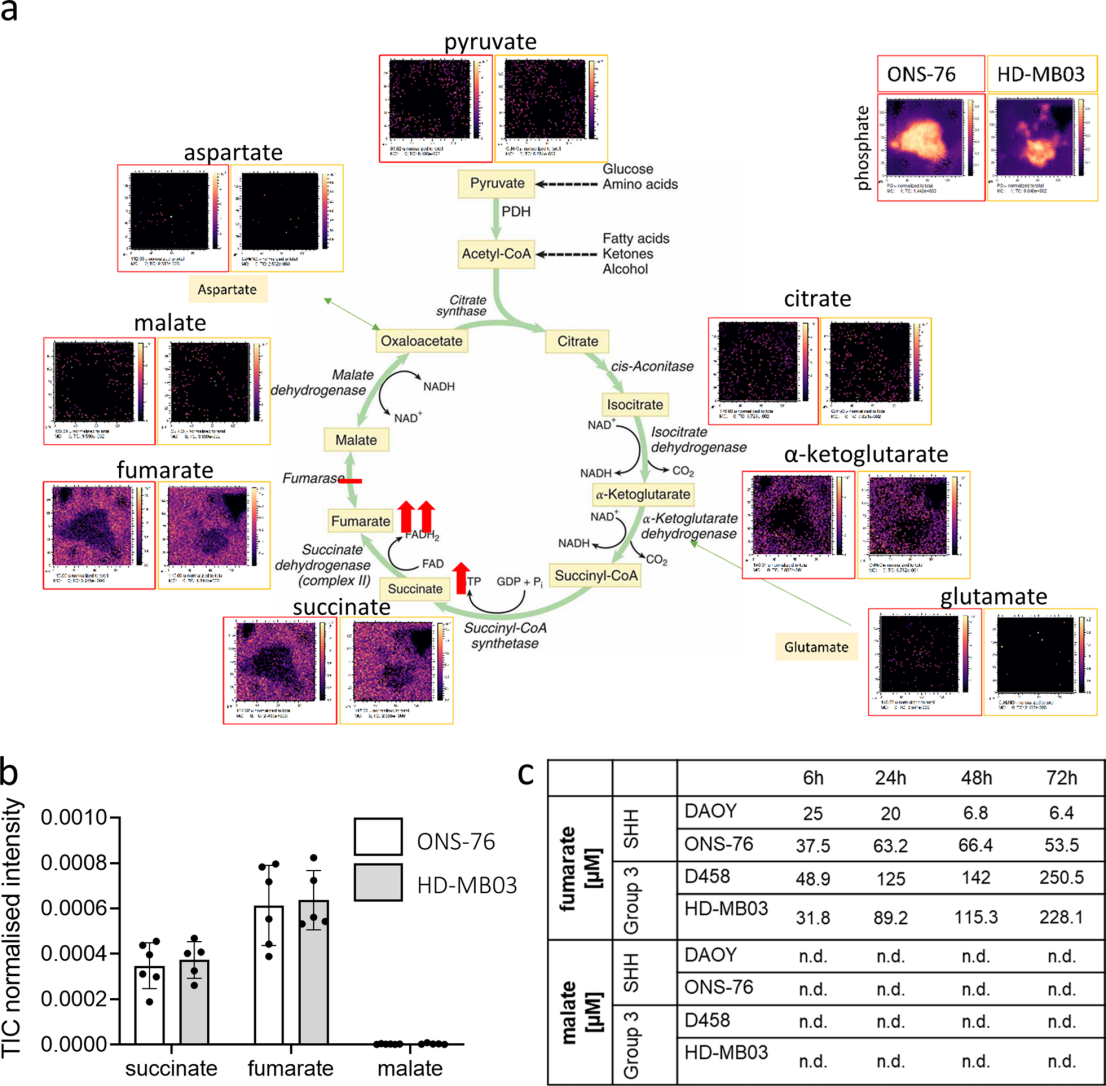
Fumarate accumulation is predominantly observed in Group 3 models. **a** 3D OrbiSIMS analysis of TCA metabolites after 16 h reveal low levels of malate (m/z 133.01), pyruvate (m/z 88.02), isocitrate (m/z 174.02) and glutamate (m/z 146.05), intermediate levels of α-ketoglutarate (m/z 145.01), high levels of succinate (m/z 117.02) and very high levels of fumarate (m/z 115.00). **b** Normalised intensity measurements confirm the accumulation of succinate and fumarate and the concurrent lack of malate after 16 h in ONS-76 and HD-MB03 cells. **c** Gel supernatants of 3 week-old SHH (DAOY, ONS 76) and Group 3 (D458, HD MB03) nodules were collected 6, 24, 48 and 72 h after medium change to quantify the secretion levels of fumarate and malate. Note the increasingly higher fumarate levels in the group 3 nodules compared to SHH nodules over time. Malate levels were below the assay detection limit at all time points (mean; *n* = 3)

The upregulation of metabolic pathways involved in glycolysis and the TCA cycle, observed in Group 3 MB by scRNAseq analysis, then led us to interrogate all TCA cell cycle metabolites in the 3D OrbiSIMS mass spectrometry imaging data (Fig. 5a, b, Additional file 2: Fig. S9a) with the aim of identifying key players in Group 3 metabolism. While detected levels of pyruvate, isocitrate and glutamate were very low to low, intermediate levels of α-ketoglutarate, high levels of succinate and very high levels of fumarate were indicated (Fig. 5a, b). Importantly, malate appears to be absent in the model. This is believed to be an accurate representation of the chemistry of these systems (as opposed to a low ionizability of malate) since the 3D OrbiSIMS data acquired in a series of control experiments demonstrated that malate could be detected at concentrations as low as 10 nM in control gels (Additional file 2: Fig. S9b–e). Using an enzymatic detection assay approach, we then confirmed that increasing concentrations of fumarate are secreted by the MB hydrogel models with at least 5-times higher concentrations in the Group 3 model while malate was again completely absent within the assay’s detection limit (Fig. 5c). This indicates an accumulation of fumarate as an onco-metabolite. In other cancers fumarate accumulation has been shown to initiate protein succination in the cytosol [30]. Targets known to be upregulated as a result of enhanced succination include oxidative stress response and epithelial-to-mesenchymal transition markers [31–34]. Notably, several of these known targets (NRF2/*NFE2L2*, *HIF1A*, *FOXM1*, *PDK1*, *ZEB1* and *TWIST1*) are significantly upregulated in MB patients compared to normal cerebellum (Additional file 2: Fig. S10 a–f).

**Fig. 6:**
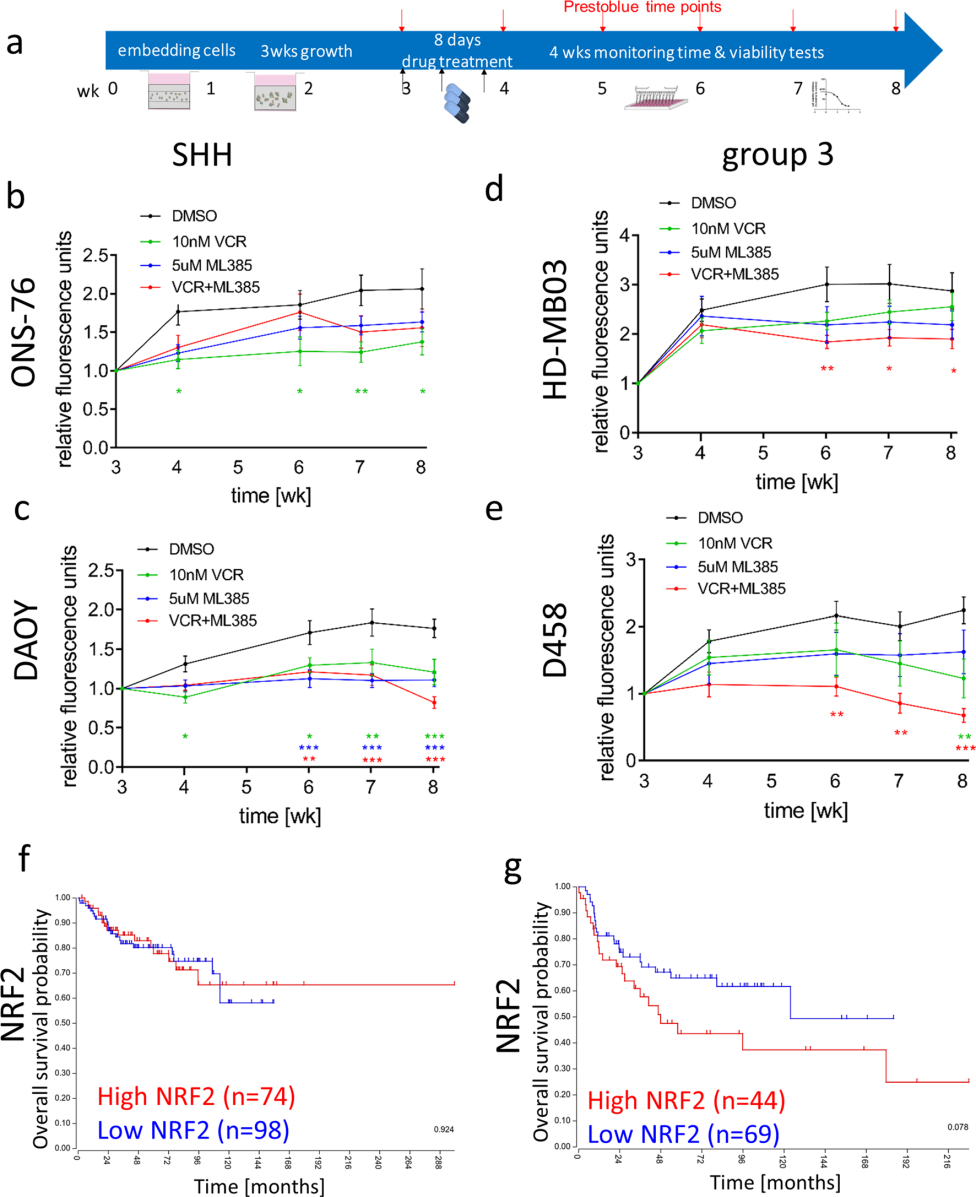
A combination of chemotherapy and NRF2 inhibition significantly improves long-term treatment of Group 3 models. **a** A scheme illustrates the course of the long-term drug treatment assay as previously established [8]. After 3 weeks of growth inside the HA hydrogels the SHH cell lines ONS-76 **b**, DAOY **c** and the Group 3 cell lines HD-MB-03 **d**, D458 **e** were treated four times with either 10 nM vincristine (green), 5 µM ML385 (NRF2 inhibitor; blue) or a combination (red) or vehicle (black) during one week and cell viability was monitored for the following four weeks in the absence of drug/vehicle present anymore. In the p53 wt SHH cell line ONS-76 only the chemotherapeutic reagent vincristine significantly decreases cell viability, while the NRF2 inhibitor (ML385) alone and in combination with vincristine are also effective in the p53mut SHH cell line DAOY. In contrast, in both Group 3 models the combination of vincristine and NRF2 inhibitor significantly reduced cell viability. (mean ± SEM, *n* = 3; Two-way ANOVA and Dunnett’s post hoc test, *P < 0.05, **P < 0.01 and ***P < 0.001 all relative to DMSO according to colour code). Analysis of the biggest publicly available MB data base [21] shows that *NRF2* (gene name: *NFE2L2*) gene expression does not predict survival of SHH (**f**; logrank test, *p* = 0.924) patients, but is associated with worse survival in Group 3 patients (**g**; logrank test, *p* = 0.078). Note the exclusive effect of *NRF2* expression in Group 3 patients

These data suggest that the accumulation of fumarate and subsequent upregulation of target genes, including succination-inducible nuclear factor erythroid 2-related factor 2 (NRF2/*NFE2L2*), might be important cancer-driving mechanisms in Group 3 MB pathogenesis. This led us to hypothesise that pre-activation of oxidative stress response pathways in Group 3 MB could contribute to chemoresistance. To test this hypothesis, three-week hydrogel culture models were treated with 4 repeated doses of vincristine alone or in combination with an NRF2 inhibitor (ML385) over one week and cell viability was monitored over the following four weeks (Fig. 6a). We have previously shown that, in common with patients, Group 3 MB models are more resistant to chemotherapy than SHH models [8]. Here again, while vincristine alone was not sufficient to significantly decrease cell viability of the two Group 3 models, combination with the NRF2 inhibitor significantly enhanced the chemotherapy effect (Fig. 6b, c). In contrast, cell viability of the p53 wildtype ONS-76 SHH model was significantly decreased by vincristine. The NRF2 inhibitor also showed a modest decrease in growth (Fig. 6d). In contrast, the NRF2 inhibitor exhibited a comparable but not additional effect to vincristine on cell viability in the p53 mutant DAOY SHH model (Fig. 6e). NRF2 gene expression is upregulated in p53 mutated patients and cell lines independent of fumarate, thus explaining the effect of the NRF2 inhibitor on the DAOY cells (Additional file 2: Fig. S10g–h). In further support of this finding, whilst statistical significance is not quite reached, gene expression levels of NRF2 appear to better distinguish between patients with good and poor prognosis in Group 3 MB but not in SHH patients (*p* = 0.078 versus *p* = 0.924, Fig. 6f, g), although NRF2 gene expression is highest in the SHH alpha subtype which consists of mostly p53 mutated patients (Additional file 2: Fig. S10g–h; [21]).

### Fumarate and lumican are biomarkers that can predict outcome in aggressive Group 3 and SHH MB

**Fig. 7:**
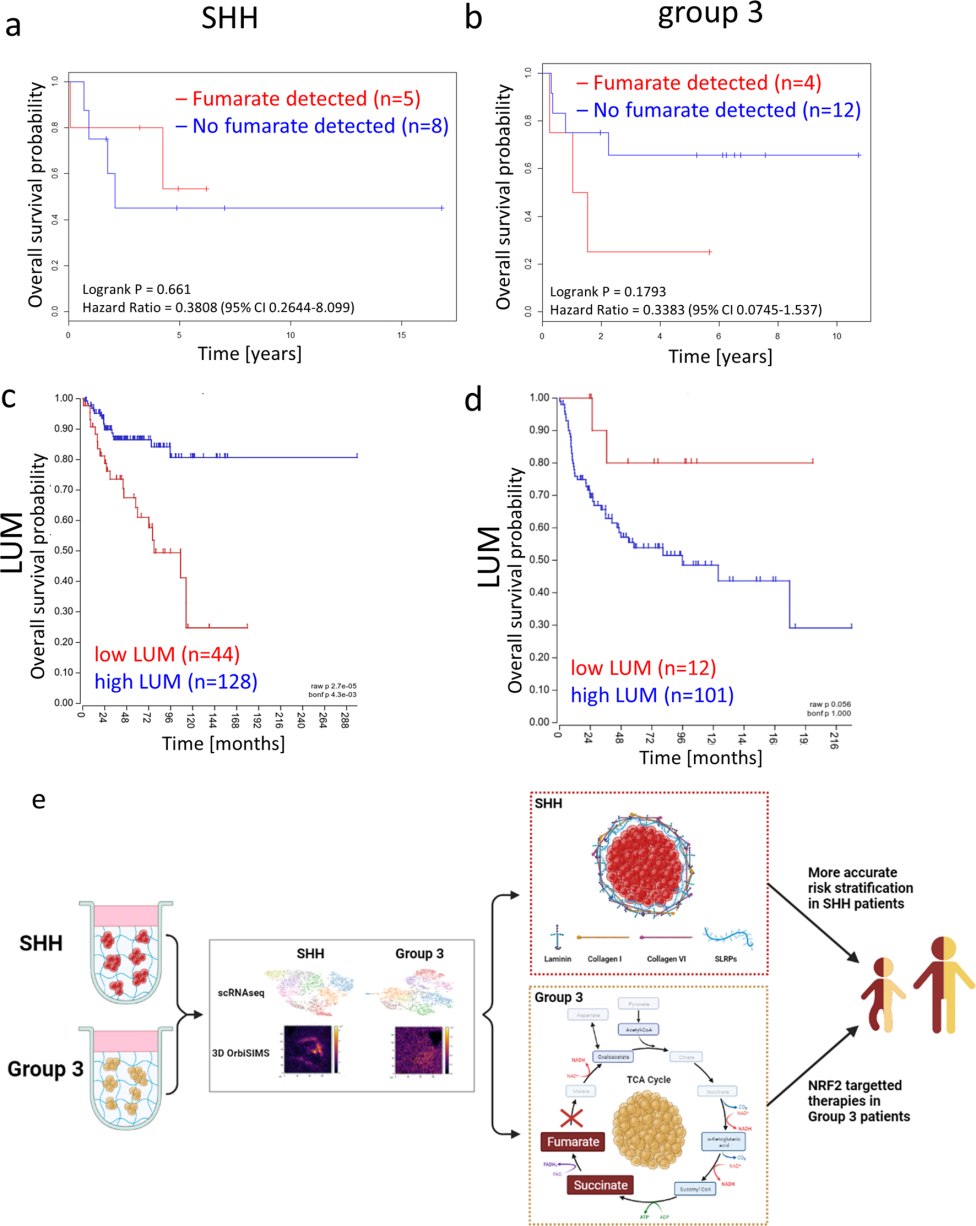
HR-MAS NMR spectroscopy detected fumarate levels group 3 and high *LUM* gene expression levels in SHH MB patients predict better overall survival. HR-MAS NMR spectroscopy analysis of fumarate in SHH (**a**, *n* = 13) and Group 3 (**b**, *n* = 16) patients indicates no survival difference for SHH patients (no fumarate detected *n* = 8; fumarate detected *n* = 5) but a strong trend for worse overall survival in Group 3 patients (no fumarate detected *n* = 12; fumarate detected *n* = 4) with detectable fumarate levels. **c** Genomic analysis of *LUM* expression in SHH (**c**; logrank test, *p* < 0.001) and Group 3 (**d**; logrank test, *p* = 0.056) patients identified that high *LUM* expression correlates with better overall survival than low *LUM* expression, whereas the opposite trend is true in Group 3 patients. **e** A graphical abstract of this study illustrating that by using by using a combination of state-of-the-art techniques (scRNAseq and 3D OrbiSIMS), in a realistic 3D model, we have identified sub-group-specific tumour phenotypes in SHH and Group 3 medulloblastoma. In the SHH sub-group we have demonstrated the formation of an ECM shell-like structure composed of laminin, type I- and VI-collagens and lumican that can be used to identify low-risk SHH tumours. This could facilitate more accurate risk stratification of SHH medulloblastoma. In the Group 3 sub-group we have shown that fumarate accumulation identifies very-high risk patients that could benefit from a chemotherapy combination with NRF2 targeted therapy approaches

Having shown that accumulation of the TCA cycle metabolite fumarate is associated with worse survival of Group 3 MB patients, and that blocking NRF2, a fumarate-mediated oxidative stress pathway member, can increase the efficacy of chemotherapeutic agents in our model, we then sought to corroborate these findings with patient data. HR-MAS NMR spectroscopy was used to detect fumarate levels in 29 SHH and Group 3 MB patient samples and the cohort was stratified into samples with no quantifiable fumarate and those with detectable fumarate levels. The presence of detectable fumarate was neither associated with metastatic disease (*p* = 0.298), large cell anaplastic histology (*p* = 1) nor MYC oncogene amplification (*p* = 1). When we analysed overall survival (OS) data, however, we found a strong trend between fumarate detection and poor outcome in Group 3 MB patients compared to no association in SHH patients (*p* = 0.1793 versus *p* = 0.661, Fig. 7a, b).

Based on our findings that SHH nodules form a sub-group specific ECM shell-like structure, we then sought to establish whether these newly identified ECM shell components could predict survival of SHH patients. Analysis of a large genomic patient dataset revealed that the SLRPs lumican, fibromodulin and decorin as well as *COL1A1* and *COL6A1* all correlate with good overall survival in SHH MB patients; suggesting that the presence of this ECM shell in SHH tumours may be a reliable biomarker of good outcome (Fig. 7c, Additional file 2: Fig. S11a and b) [21]. Notably, in Group 3 patients lumican expression is associated with a much poorer survival, highlighting that capturing lumican as part of a complex with other SLRPs and *COL1A1* and *COL6A1* in an ECM ‘shell’is key to this effect on outcome in the SHH sub-group (Fig. 7d).

## Discussion

In this study we have demonstrated, using single cell RNA Seq and high mass resolved metabolic imaging, that biological differences between medulloblastoma molecular subgroups also exist at the level of ECM structure and cellular metabolites. Importantly, these differences can be used to identify clinically-relevant targetable differences between subgroups.

Our study identified relevant extracellular matrix factors and adhesion pathways in SHH subpopulations, while extensive TCA cycle activation and in particular fumarate accumulation is a feature of Group 3 medulloblastoma. Fumarate accumulation is well described as a driver of tumour aggressiveness in renal cancers that are fumarate hydratase-deficient, due to loss of heterozygosity of the enzyme or upregulation of the mammalian target of rapamycin (mTOR) [35–37]. As a consequence of high intracellular fumarate levels, fumarate can react with the sulphydryl moiety of cysteine residues to produce 2-succinyl-cysteine, known as protein succination [38]. Protein succination can modify the activity of several cysteine-containing proteins, including the NRF2 inhibitor Kelch-like ECH-associated protein 1 (KEAP1) thus activating pathways involved in epithelial-to-mesenchymal-transition as well as oxidative stress responses [31–34]. Since oxidative stress response pathways are activated despite normal oxygen conditions this phenomenon has been described as a pseudo-hypoxic state [39]. Several chemotherapeutic reagents, including vincristine, are known to induce oxidative stress [40–42]. Therefore, the permanent upregulation of electrophilic and oxidative stress response pathways, for example mediated by the master regulator NRF2, can cause chemoresistance in several cancer types [43–46]. NRF2 inhibition or knockdown restored chemosensitivity in breast, colorectal, pancreatic and gallbladder cancer and is being increasingly discussed as a novel chemotherapy combination strategy [47–49]. In glioma, the most aggressive type of brain tumour in adults, overall survival as well as disease-free survival were significantly shorter in patients with high NRF2 expression and NRF2 expression also correlated with tumour grade [50].

Our study highlights that a similar NRF2-mediated pre-activation of oxidative stress response pathways could cause chemoresistance and impair treatment of the most aggressive MB subgroup. Interruption of NRF2 signalling could therefore become an interesting new target for MB therapy, especially in combination with existing chemotherapy regimens. In addition, NRF2 inhibitors could be beneficial in p53 mutated SHH cases. Although we did not detect increased levels of fumarate in the p53 mutated SHH model, NRF2 gene expression is elevated as result of the p53 mutation as described in other cancer types [51, 52]. Therefore, future studies, including in vivo studies, will be needed to investigate whether Group 3 and p53 mutated SHH MB tumour treatment can be improved by NRF2 inhibitors.

Apart from effects on NRF2, it is important to mention that protein succination causes multiple, complex intracellular changes. Therefore, broader therapy approaches that prevent fumarate accumulation inside cancer cells might be more efficient in Group 3 MB. In addition to new therapy options, fumarate levels also need to be tested as potential markers for patient prognosis and indicators of therapy failure. In our study, we detected fumarate levels in tumour samples of Group 3 patients at first resection before chemotherapy and showed a strong trend for poorer overall survival in these patients. Future larger studies should include the measurement of fumarate levels before and after treatment to test its potential as a prognostic marker for aggressive Group 3 MB.

In addition, the combination of single cell sequencing and mass spectrometry imaging techniques identified a specific ECM phenotype in SHH tumour models. Recently, we showed that laminin is a core component of the SHH shell-like structure and that laminin expression predicts good overall survival for SHH patients [8]. Here we show that collagens and sulphur-containing species are also part of this shell -like structure. SHH tumours are highly heterogeneous ranging from very high risk p53 mutated tumours to low risk desmoplastic tumours found in infants [53], in addition to standard risk tumours with a classical phenotype. The shell-like structure that we observe may be a more subtle form of nodularity, not detectable in standard histological analyses, but able to predict good outcome in tumours that would otherwise be classified as standard risk classical tumours.

Through the analysis of genomic patient data and immunohistochemical staining of SHH nodules grown in hydrogels, we have demonstrated that these sulphur-containing species are most probably a combination of SLRPs. Our data supports the proposal that lumican is one of these SLRPs, since it is located in the shell structure. Lumican is a proteoglycan that can contain multiple KS chains and plays a crucial role in collagen fibrillogenesis. It therefore may facilitate the formation and organisation of the collagen component of the SHH shell-like structure [27–29]. In addition, survival analysis of the same patient dataset revealed that lumican (plus fibromodulin and decorin) as well as *COL1A1* and *COL6A1* predict good overall survival for SHH patients in the same manner as laminin, further suggesting that they are all part of the same ECM shell-like structure as depicted in Fig. 7 (Fig. 7c and e, Additional file 2: Fig. S11a–d). In fact, during the course of this study, lumican, COL1A1 and COL6A1 have all been validated independently by Trombetta-Lima et al. as components of the medulloblastoma secreted extracellular matrix, by proteomic and immunohistochemical analysis of patient tumours [54]. Furthermore, the expression of lumican has previously been shown to have a restrictive role in the progression of several other cancer types as well as being correlated with better survival, illustrating the potential anti-tumorigenic effect of this ECM shell-like structure on SHH MB [55–61].

## Conclusions

This study highlights that a combination of state-of-the-art single cell sequencing and mass spectrometry imaging techniques in a realistic 3D model allows the identification of sub-group-specific tumour cell populations whose unique functions can be exploited for future prognostic and therapeutic strategies. In this first example of such an approach, we show that fumarate accumulation in aggressive Group 3 MB tumours identifies very-high risk patients, potentially identifiable using HR-MAS NMR spectroscopy, who could benefit from a chemotherapy combination with NRF2 targeted therapy approaches. Conversely, we also demonstrate that low-risk SHH MB tumours can be identified by the formation of an ECM shell-like structure composed of laminin, type I- and VI-collagens and lumican; these patients may benefit from less harsh treatment regimens in order to ensure the best possible quality of life post-treatment. Patients without these ECM proteins could be classed as high-risk and may require more intensive treatment.

In paediatric cancer a crucial aim is reliable risk stratification including individual therapy approaches to maximise therapy effects while minimising quality of life reducing long-term side effects. Therefore, we recommend including fumarate and a panel of the SHH ECM shell proteins in future trials to test if SHH and Group 3 patients can be stratified based on these biomarkers for personalized treatments.

## Supplementary Information

**Additional file 1.** scRNAseq data ONS76 HD-MB 03 - ALL SIGNIFICANT GENES PER CLUSTER. This is and Excel spreadsheet that contains data relating to all the significantly altered genes with their associated Log2 fold change and *p*-values.

**Additional file 2. **Supplementary Figures and Methods. This is a word file that contains additional figures and their legends, alongside additional methods and references relating to these.

## Data Availability

All data generated or analysed during this study are included within the article (and its Additional Information files). The scRNA and RNA sequencing data have been deposited in the ArrayExpress database at EMBL-EBI (www.ebi.ac.uk/arrayexpress) under accession number E-MTAB-12591 and E-MTAB-9823 respectively. Genes differentially expressed between clusters can be found in Additional File 1.

## Abbreviations

MB: Medulloblastoma
SLRPs: Small leucine-rich proteoglycans
TCA: Tricarboxylic acid
scRNAseq: Single cell RNA sequencing
NRF2: Nuclear Factor Erythroid 2-Related Factor 2
SHH: Sonic hedgehog
ECM: Extracellular matrix
3D OrbiSIMS: A Time of Flight Secondary Ion Mass Spectrometer (ToF–SIMS) with hybrid OrbiTrapTM functionality
KS: Keratan sulfate
