# Supplementary material for: Identifying new biomarkers of aggressive Group 3 and SHH medulloblastoma using 3D hydrogel models, single cell RNA sequencing and 3D OrbiSIMS imaging
Source: Acta Neuropathol Commun. 2023 Jan 11;11:6. doi: 10.1186/s40478-022-01496-4 (PMC9835248; doi:10.1186/s40478-022-01496-4)
Supplement: Supplementary file 2 — Additional file 2. Supplementary Figures and Methods. This is a word file that contains additional figures and their legends, alongside additional methods and references relating to these. [file 40478_2022_1496_MOESM2_ESM.docx]

# Supplementary Methods and Figures

# Identifying new biomarkers of aggressive Group 3 and SHH medulloblastoma using 3D hydrogel models, single cell RNA sequencing and 3D OrbiSIMS mass spectrometry imaging

**Authors**

Franziska Linke^1^, James E. C. Johnson^1^, Stefanie Kern^2^, Christopher D. Bennett^3,4^, Anbarasu Lourdusamy^1^, Daniel Lea^5^, Steven C. Clifford^6^, Cathy L. R. Merry^7^, Snow Stolnik^2^, Morgan R. Alexander^2^, Andrew C. Peet^3,4^ David J. Scurr^2^, Rian L. Griffiths^2^, Anna M. Grabowska^7^, Ian D. Kerr^8^, Beth Coyle^1^

^1^Children’s Brain Tumour Research Centre, School of Medicine, Biodiscovery Institute, University of Nottingham, Nottingham, UK

^2^School of Pharmacy, University of Nottingham, Nottingham, UK

^3^Institute of Cancer and Genomic Sciences, University of Birmingham, Birmingham, UK

^4^Birmingham Children's Hospital, Birmingham, UK

^5^Digital Research Service, University of Nottingham, Nottingham, UK

^6^ Newcastle University Centre for Cancer, Translational & Clinical Research Institute, Wolfson Childhood Cancer Research Centre, Herschel Building, Level 6, Brewery Lane, Newcastle upon Tyne, NE1 7RU

^7^Stem Cell Glycobiology Group, Biodiscovery Institute, University of Nottingham, Nottingham, UK

^8^School of Life Sciences, University of Nottingham, Nottingham, UK

## Supplementary Figures

Supplementary Table 1: Overview of cluster characteristics

| Cluster | Cell number | Upregulated genes | Downregulated genes | Ratio |
| --- | --- | --- | --- | --- |
| O1 | 761 | 133 | 309 | 0.43 |
| O2 | 568 | 33 | 5 | 6.6 |
| O3 | 567 | 22 | 125 | 0.18 |
| O4 | 471 | 12 | 0 | n.a |
| O5 | 456 | 108 | 61 | 1.77 |
| O6* | 447 | 1 | 0 | n.a |
| O7 | 422 | 334 | 150 | 2.23 |
| O8 | 397 | 93 | 206 | 0.45 |
| O9 | 359 | 141 | 306 | 0.46 |
| Total ONS | 4448 | 877  (656) | 1162  (720) | 0.75  (0.9) |
| H1 | 722 | 768 | 114 | 6.74 |
| H2 | 606 | 9 | 1246 | 0.01 |
| H3 | 557 | 938 | 75 | 12.5 |
| H4 | 515 | 13 | 0 | n.a |
| H5* | 478 | 0 | 0 | n.a |
| H6 | 373 | 165 | 505 | 0.33 |
| H7 | 370 | 171 | 0 | n.a |
| H8 | 300 | 4 | 149 | 0.03 |
| H9 | 227 | 28 | 217 | 0.13 |
| Total HD | 4148 | 2096  (1457) | 2306  (1397) | 0.91  (1) |


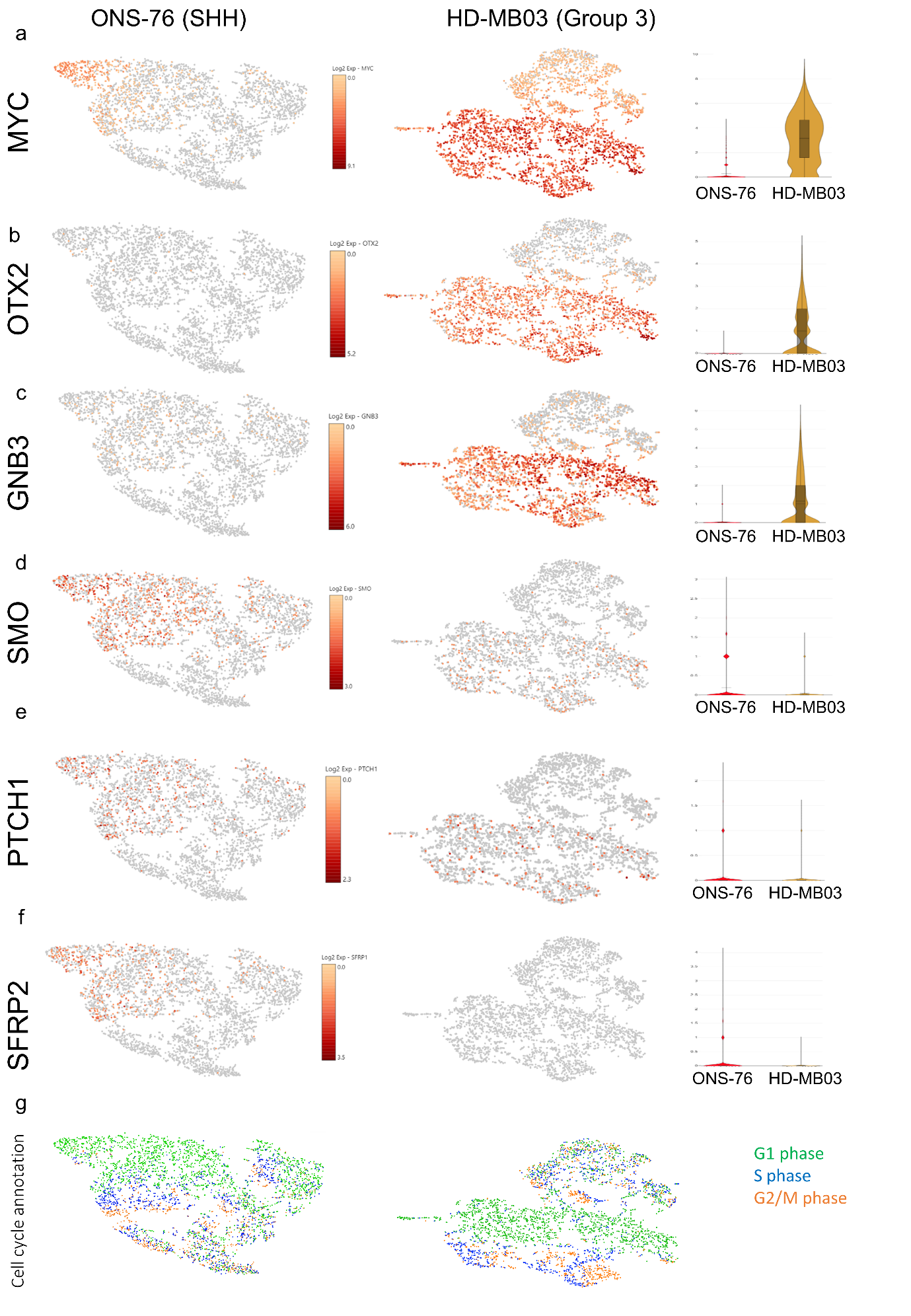


**Supplementary Figure 1: scRNAseq data shows subgroup-specific marker gene expression in SHH (ONS‑76) and Group 3 (HD‑MB03) hydrogel models.** Group 3 marker genes MYC (a), OTX2 (b) and GNB3 (c) are much higher expressed in the Group 3 hydrogel model, while SHH marker genes SMO (d), PTCH1 (e) and SFRP2 (f) are higher expressed in the SHH model. (g) t-SNE plot of cell cycle annotation visualizes the cell cycle state of each sequenced cell.


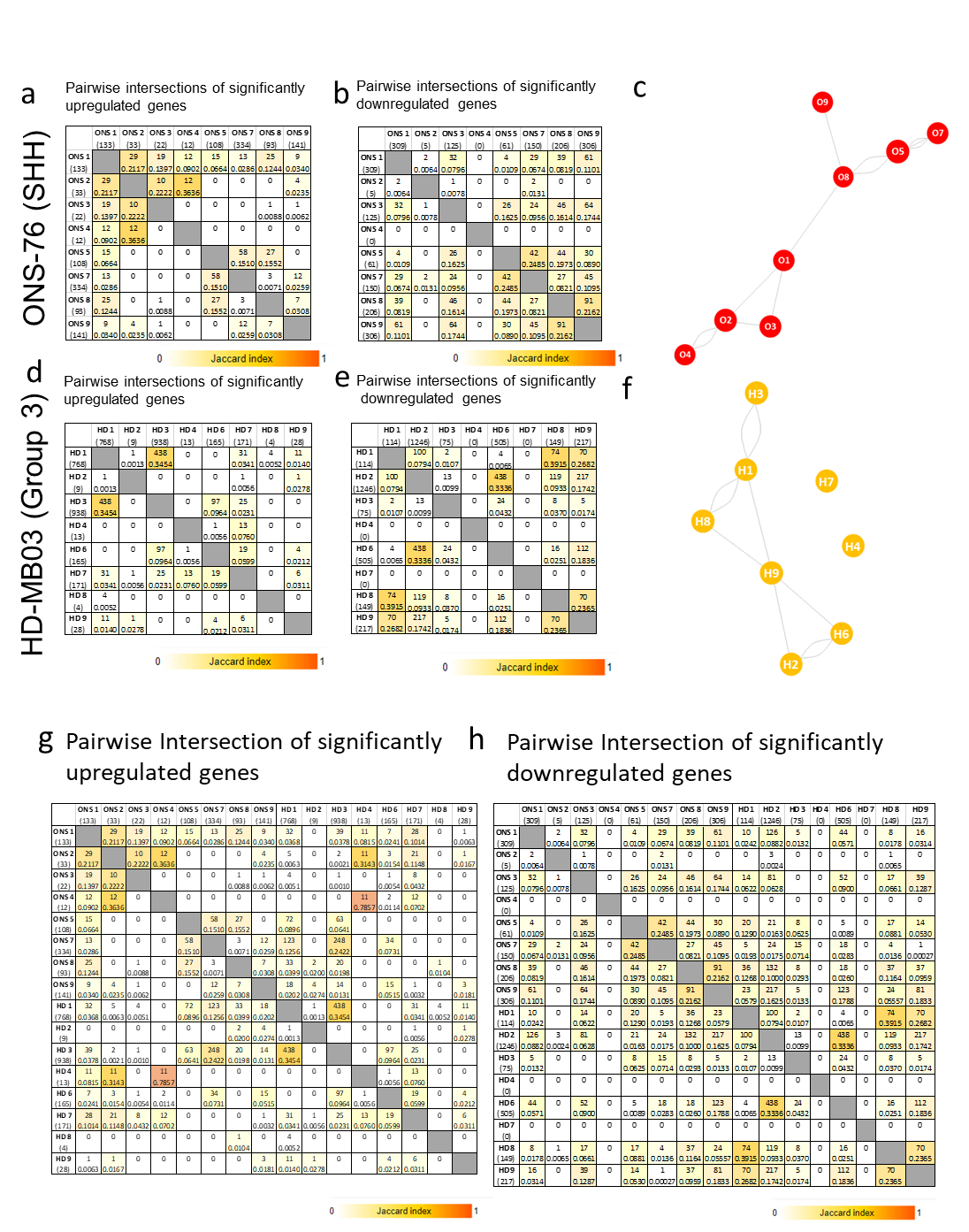


**Supplementary Figure 2: Clusters of the Group 3 and SHH model share significantly up- and downregulated genes.** Significantly up-and downregulated genes between cell clusters were used to determine and visualize cluster similarities and network connections for ONS‑76 **(a-c)** and HD‑MB03 **(d-f)** cells. (Clusters O6 and H5 were excluded from the analysis due to a lack of significantly up-and downregulated cluster-specific genes.) Heads of the intersection tables name the cluster with the total number of significantly up-or downregulated genes compared to all other clusters is given in brackets underneath the cluster name. The table states for each cluster pair the total number of shared significantly up-or downregulated genes and the corresponding Jaccard Index below. The network graph shows cluster similarity based on the mean Jaccard index of shared up-and downregulated genes with the number of connections indicating higher Jaccard values [1: 0.15<Jaccard≥0.1; 2: 0.2<Jaccard≥0.15; 3: 0.3<Jaccard≥0.2]. Significantly up- (**g**) and downregulated genes (**h**) between all ONS‑76 and HD‑MB03 cell clusters were used to determine cluster similarities (Clusters O6 and H5 were excluded from the analysis due to a lack of significantly up-and downregulated cluster-specific genes).


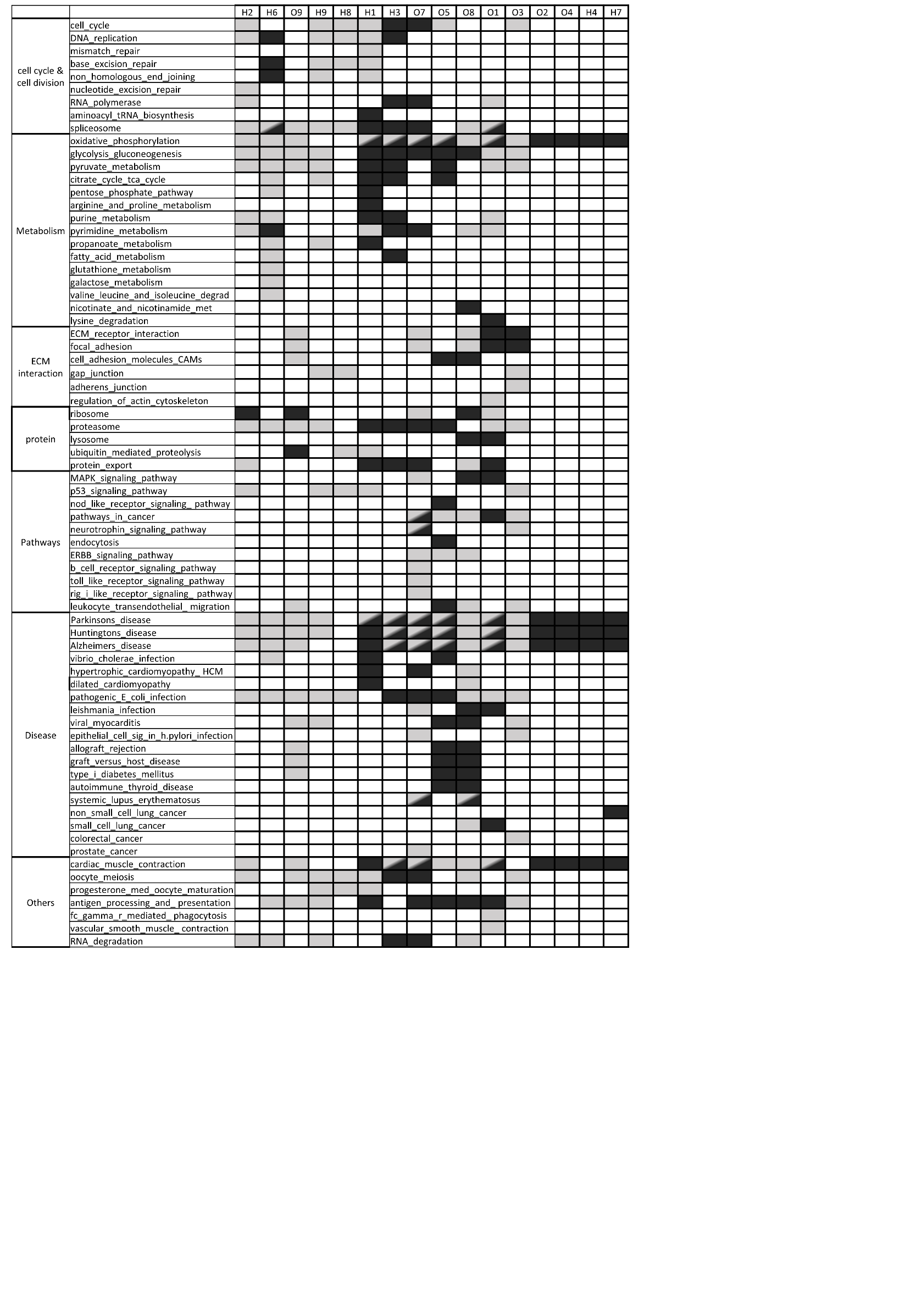


**Supplementary Figure 3: Cell clusters of SHH and Group 3 models not only share functions but also present with unique subgroup-specific cell functions.** Top20 KEGG pathways of significantly up- (dark grey) and down- (light grey) regulated genes are listed for each cluster. Note the unique presence of ECM and adhesion subpopulations in ONS 76 clusters while metabolic clusters are dominant in HD MB03.


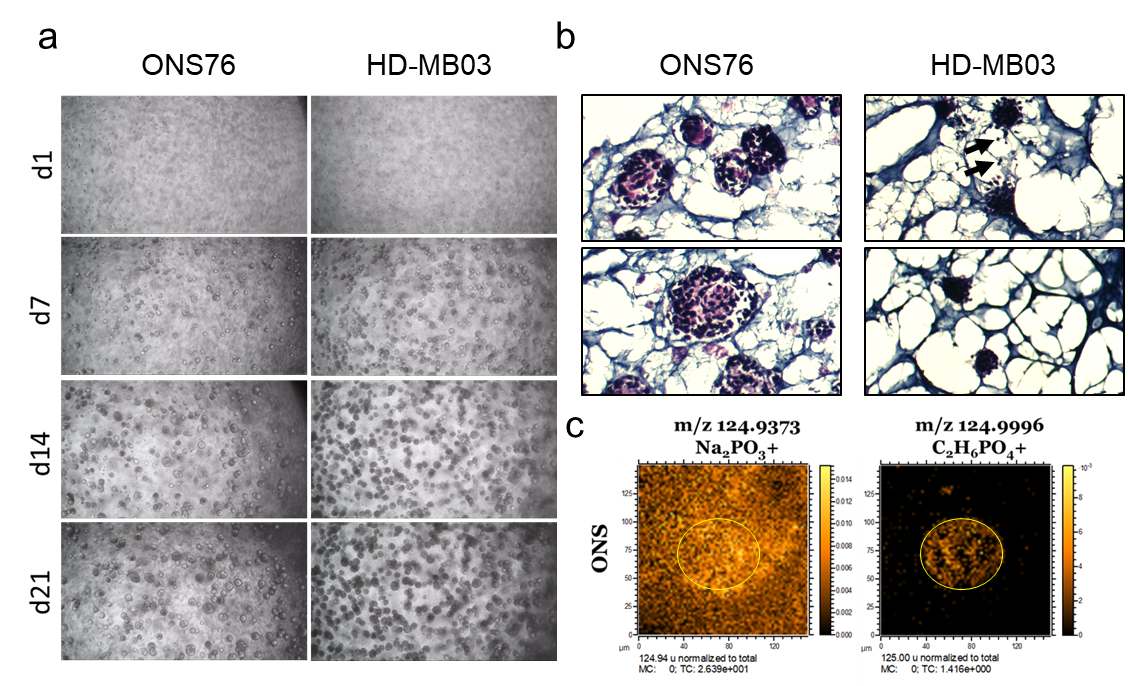


**Supplementary Figure 4: Three weeks old hydrogel models are analysed using the 3D OrbiSIMS mass spectrometry imaging.** ONS‑76 (SHH) and HD-MB03 (Group 3) cells grow for three weeks inside HA hydrogels and form nodules over time (a). H&E staining of these nodules shows looser density in SHH models and migrating cells in the Group 3 model (arrows). (c) Analysis of cryo-preserved nodule sections can be imaged using the 3D OrbiSIMS and closely related peaks can clearly be distinguished such as Na_2_PO_3_^+^ (m/z 124.9373) and C_2_H_6_PO_4_^+^ (m/z 124.9996). Yellow border indicates nodule area to help distinguishing between signals from inside the nodule and its surrounding.


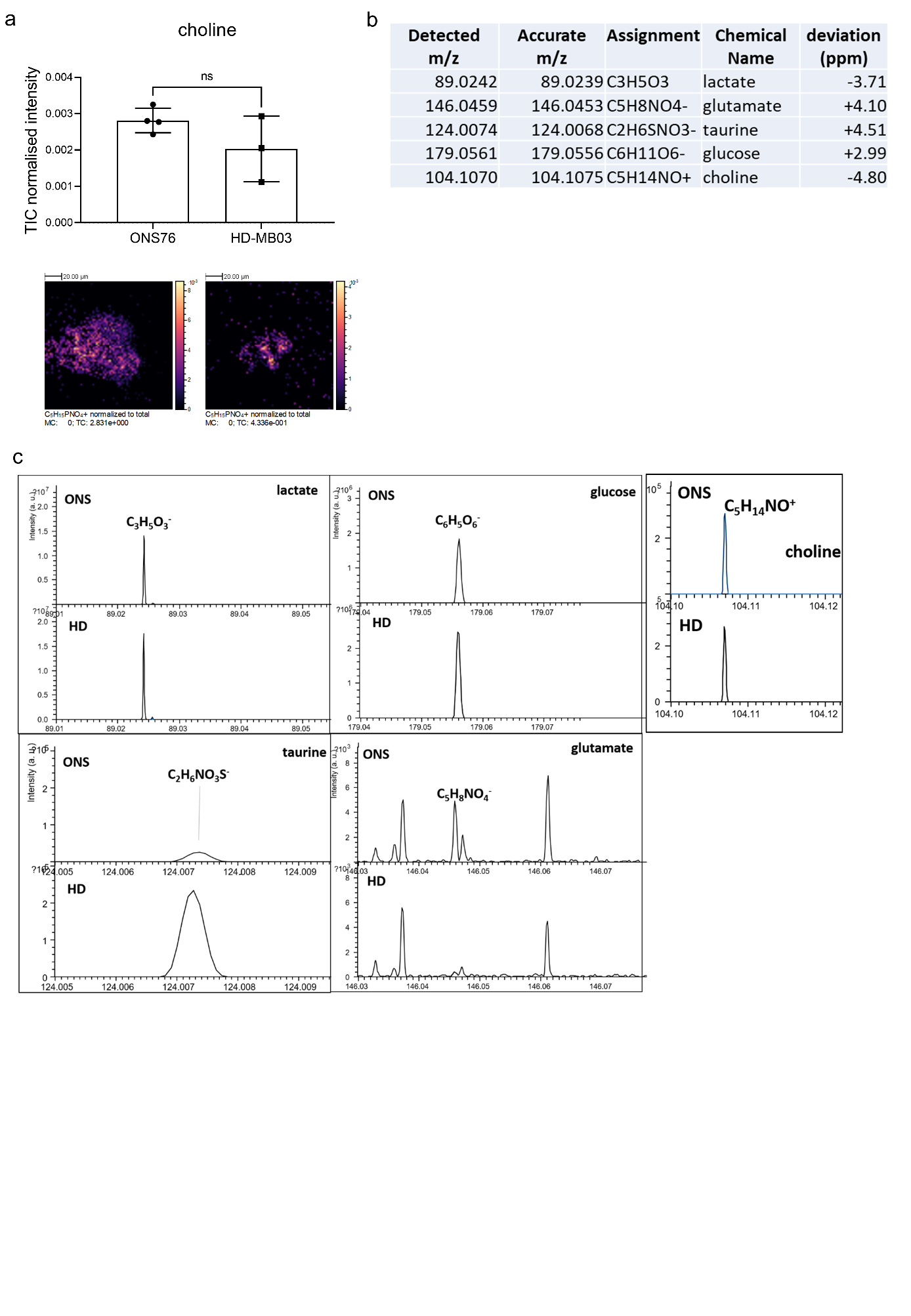


**Supplementary Figure 5:** **3D OrbiSIMS mass spectrometry imaging confirms high choline levels using positive ion mode.** **a)** Choline levels are high in SHH and Group 3 nodules (mean± SD; SHH: n=4; Group 3: n=3 unpaired t-test). b) Overview of detected and accurate m/z of lactate, glutamate, taurine, glucose and choline. c) Spectra of lactate, glutamate, taurine, glucose and choline as detected by 3D OrbiSIMS.


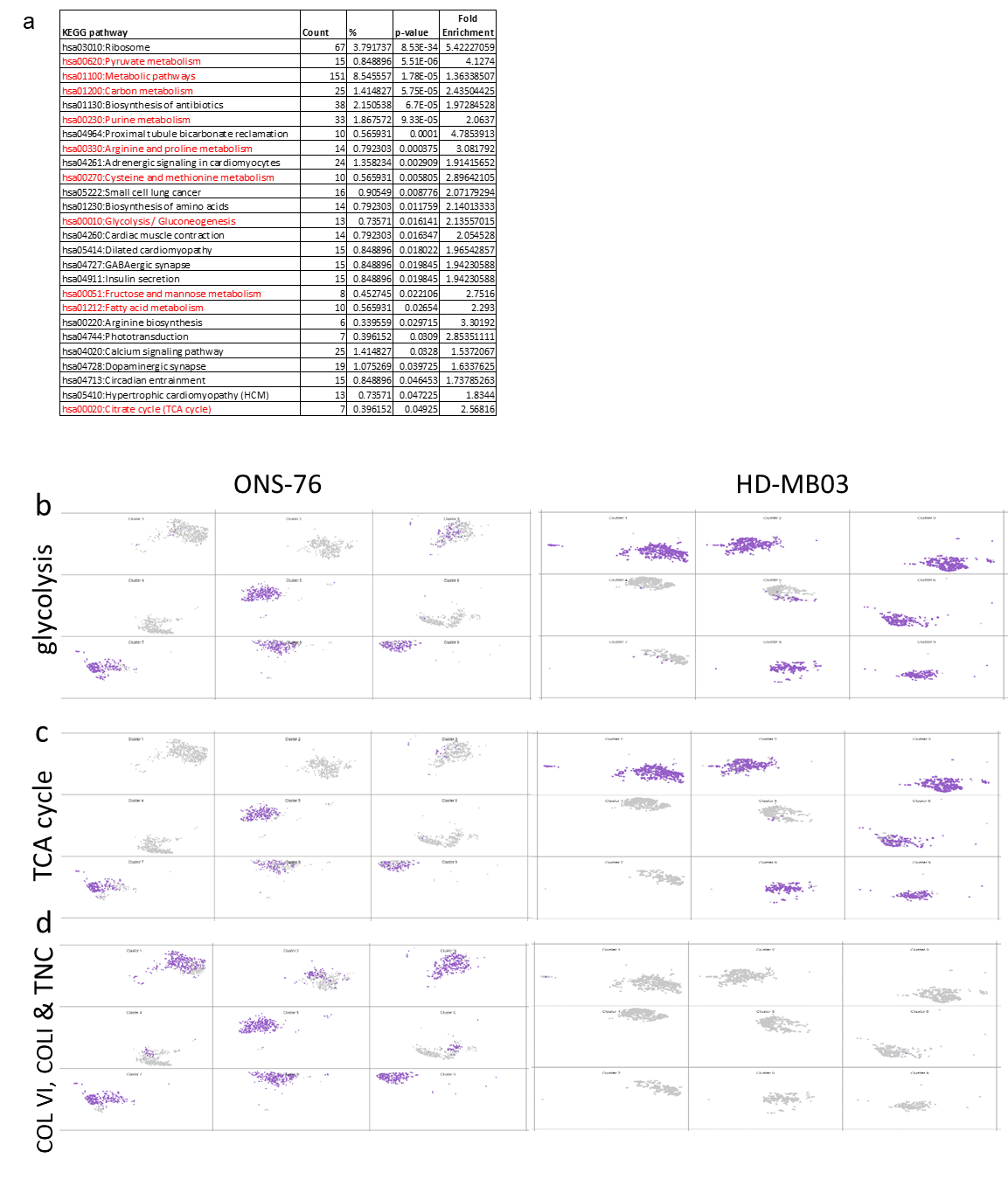


**Supplementary Figure 6: Cluster specific expression of glycolysis, TCA and ECM genes.** (**a**) Metabolic pathways including glycolysis and TCA cycle are predominantly active in Group 3 nodules. Several critical components of glycolysis and TCA cycle are significantly upregulated in the Group 3 model. Accordingly, several metabolic KEGG pathways are significantly upregulated in the Group 3 nodule (metabolic pathways in red). Gene expression for glycolysis (**b**; ALDOA, LDHB, GALM, AKR1A1, HKDC1, ENO2, ALDH2, ENO3, PGAM2, PFKM, PDHA1, PCK2, ALDH9A1), TCA cycle (**c**; SUCLG1, PDHA1, PCK2, MDH2, PC, MDH1, FH) and ECM genes (**d**; COL6A1, COL6A2, COL6A3, COL1A1, TNC) on the single cell level are displayed for the SHH (ONS 76) and Group 3 (HD MB03) model according to cluster (purple: gene expressing cell; grey: non-expressing cell).


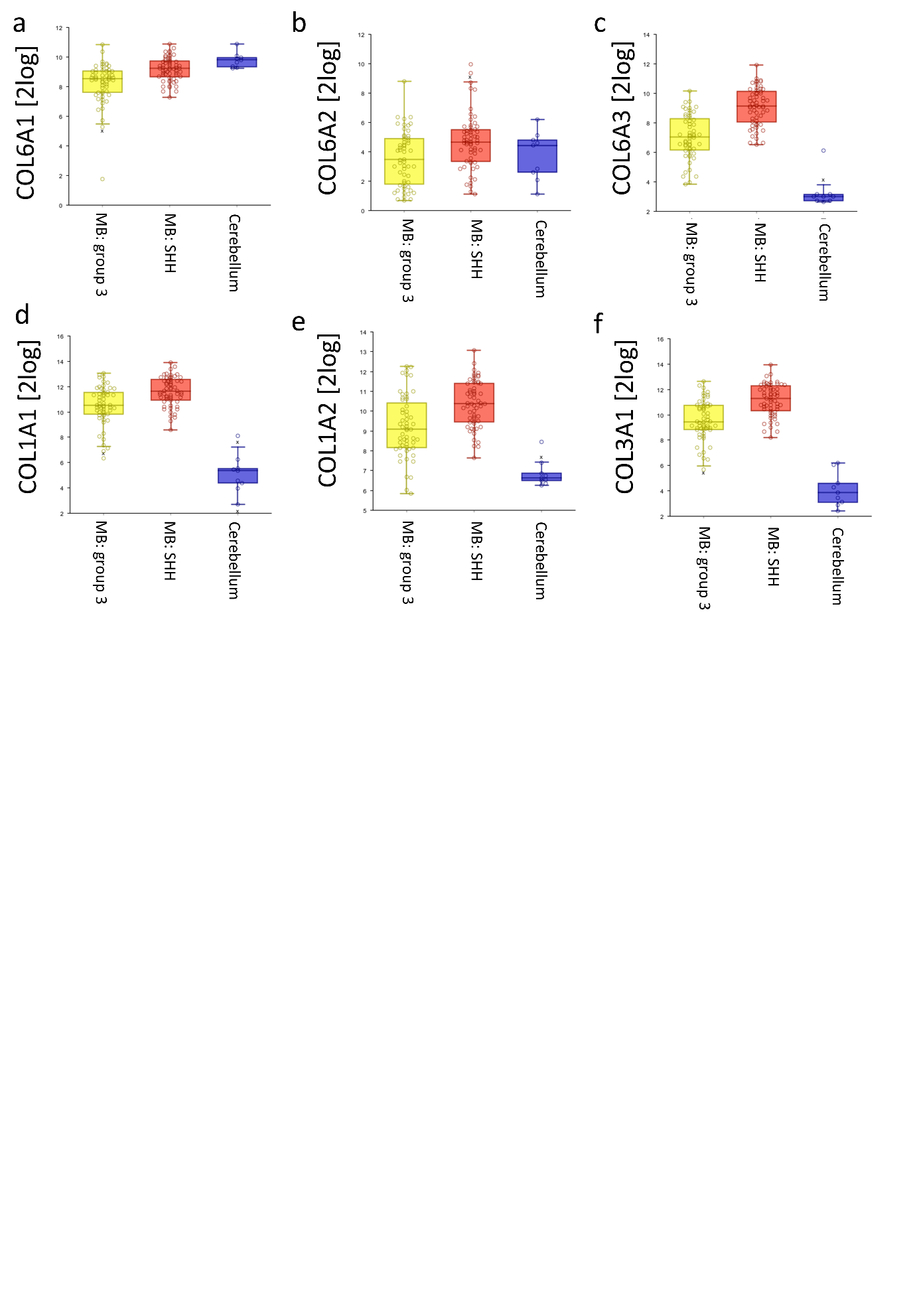


**Supplementary Figure 7:** **Expression of several ECM factors is increased in MB patients compared to normal cerebellum.** Expression of *COL6A1*, *COL6A2*, *COL6A3, COL1A1*, *COL1A2* and *COL3A1* is higher in MB patients compared to normal cerebellum using the R2: MegaSampler tool (Pfister data set [1] is compared to the 9 cerebellum samples from the Roth353 dataset [2] [GSE3526], SHH: n=59, Group 3: n=56, cerebellum: n=9).


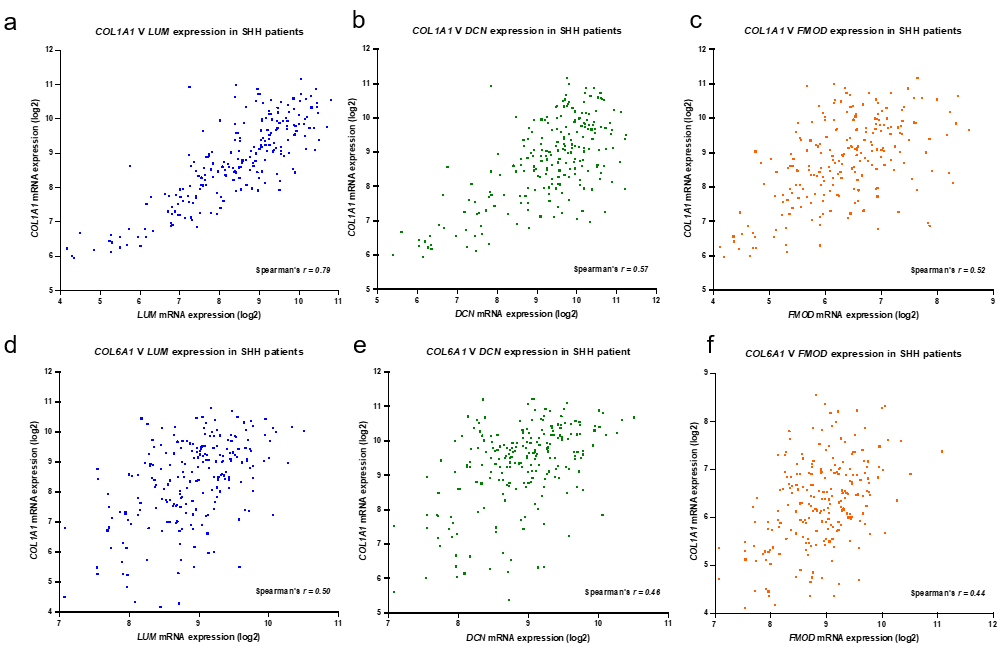


**Supplementary Figure 8: The expression of COL1A1 and COL6A1 correlates with the expression of several SLRPs in genomic SHH MB patient data**. Analysis of the biggest publicly available MB data base [23] shows that the expression of COL1A1 is positively correlated with the expression of LUM (**a**; r=0.79), DCN (**b**; r=0.57), FMOD (**c**; r=0.52). Analysis also shows that that the expression of COL1A6 is also positively correlated with the expression of LUM (**a**; r=0.50), DCN (**b**; r=0.46), FMOD (**c**; r=0.44).


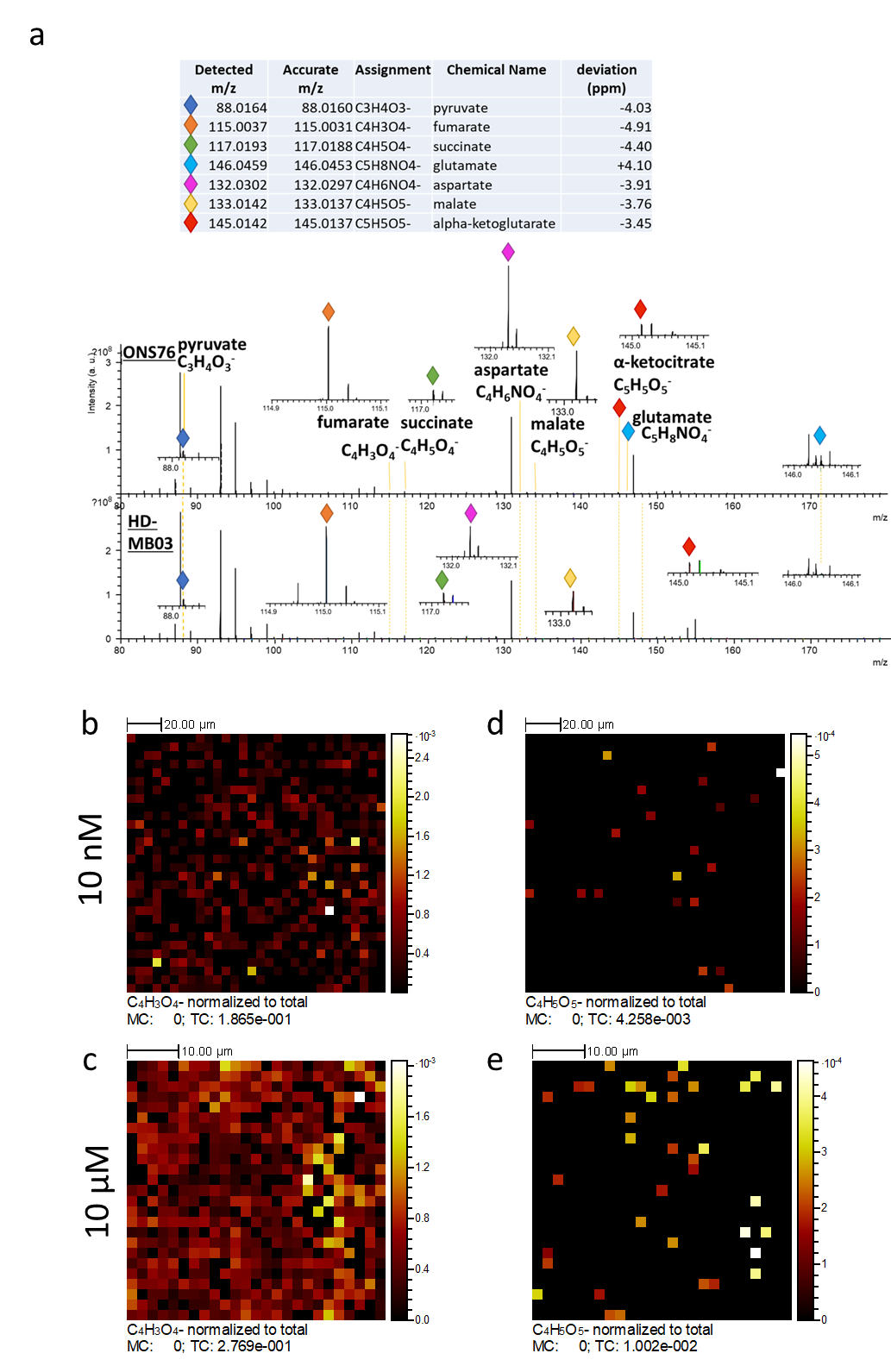


**Supplementary Figure 9: 3D OrbiSIMS mass spectrometry imaging can detect 10 nM fumarate and malate in reference gels. *(a)*** *S*pectra of TCA metabolites in ONS76 and HD-MB03 samples. Cell-free HA gels containing 10 nM or 10 µM fumarate (**b,c**; C_4_H_3_O_4_^-^, m/z 115.00) or malate (**d,e**; C_4_H_5_O_5_^-^, m/z 133.01) respectively have been measured and visualized using the 3D OrbiSIMS.


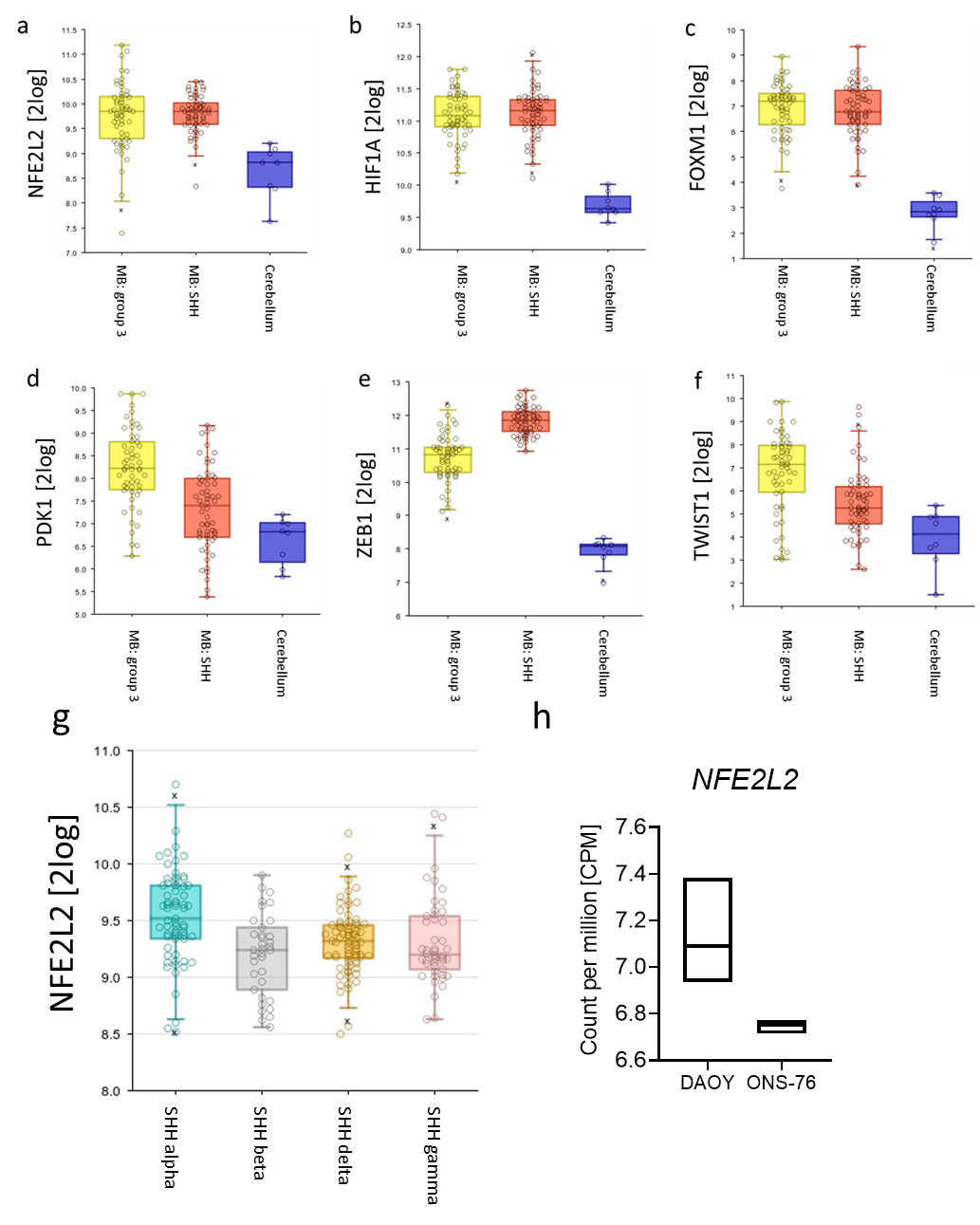


**Supplementary Figure 10: Expression of target genes of fumarate-mediated succination are increased in MB patients compared to normal cerebellum.** Expression of *NFE2L2* (**a**; NRF2), *HIF1A* (**b**), *FOXM1* (**c**), *PDK1* (**d**), *ZEB1* (**e**) and *TWIST1* (**f**) are higher in MB patients compared to normal cerebellum using the R2: MegaSampler tool (Pfister data set [1] is compared to the 9 cerebellum samples from the Roth353 dataset [2] [GSE3526], SHH: n=59, Group 3: n=56, cerebellum: n=9). (**g**) NRF2 (*NFE2L2*) gene expression is highest in the SHH alpha subtype with consists of the most p53 mutated patients (data set from Cavalli et al[3] is presented according to the subgroups SHH alpha: n=65; SHH beta: n=35; SHH delta: n=76; SHH gamma: n=47). (**h**) *NRF2* (NFE2L2) gene expression of SHH MB cell lines shows highest expression in DAOY in cell line RNA sequencing data published
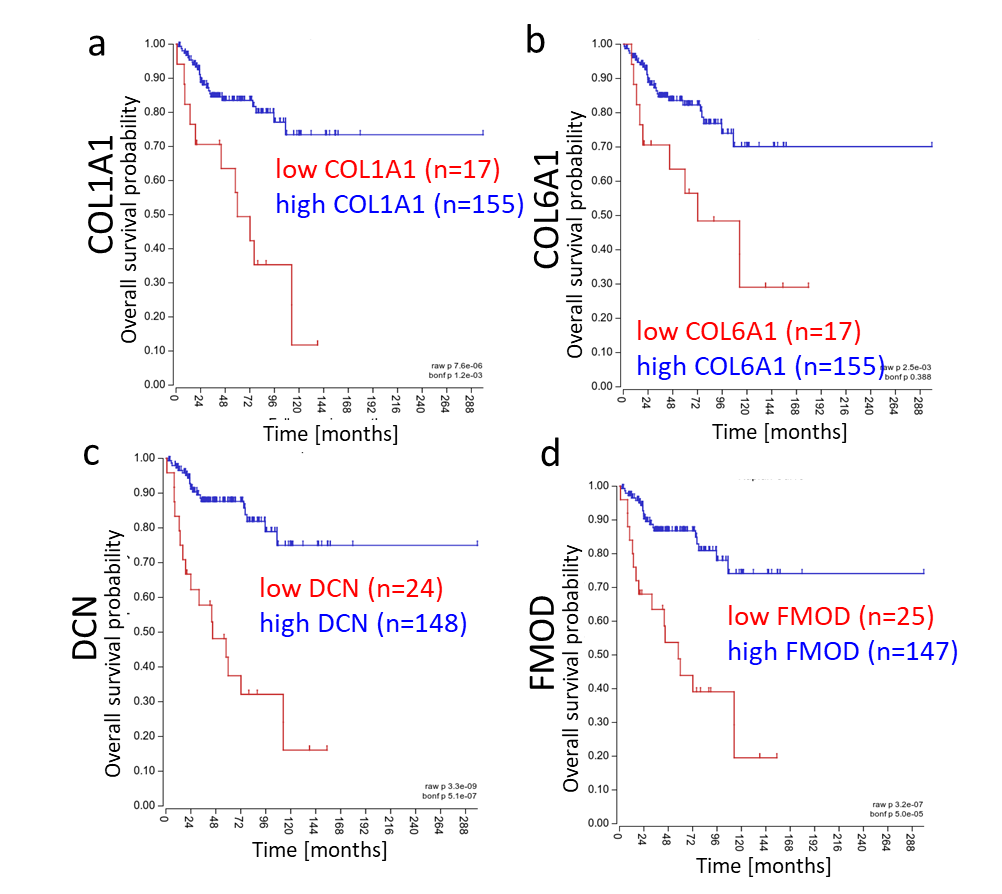
previously [4].

**Supplementary Figure 11: high COL1A1, COL6A1, DCN and FMOD gene expression levels in SHH MB patients predict better overall survival.**  Analysis of the biggest publicly available MB data base [23] shows that high gene expression of COL1A1 (**a;** logrank test, p<0.001), COL6A1 (**b;** logrank test, p=0.0025), DCN (**c;** logrank test, p<0.001) and FMOD (**d;** logrank test, p<0.001), all predict better overall survival of SHH patients than low expression.

**Supplementary Methods**

Overall gene expression comparison between HD-MB03 and ONS-76

To determine which genes were more highly expressed in either the HD-MB03 or ONS-76 sample, the median-normalised counts for each gene in each cluster were combined into an average median-normalised count for each gene. This was done for both samples and then the Wilcoxon test was used to determine significant (p < 0.05) differential expression of the genes. The Benjamini-Hochberg method was used to correct for multiple testing. In the end, significant differentially expressed genes were those with an adjusted p-value less than 0.05. Enrichment analysis of KEGG pathways was performed using DAVID Bioinformatics Resources 6.8 [5,6].

## References

1 Northcott PA, Buchhalter I, Morrissy AS, *et al.* The whole-genome landscape of medulloblastoma subtypes. *Nature* 2017; **547**: 311-317

2 Roth RB, Hevezi P, Lee J, *et al.* Gene expression analyses reveal molecular relationships among 20 regions of the human CNS. *Neurogenetics* 2006; **7**: 67-80

3 Cavalli FMG, Remke M, Rampasek L, *et al.* Intertumoral Heterogeneity within Medulloblastoma Subgroups. *Cancer Cell* 2017; **31**: 737-754.e6

4 Linke F, Aldighieri M, Lourdusamy A, *et al.* 3D hydrogels reveal medulloblastoma subgroup differences and identify extracellular matrix subtypes that predict patient outcome. *J Pathol* 2021; **253**: 326-338

5 Huang DW, Sherman BT, Lempicki RA. Systematic and integrative analysis of large gene lists using DAVID bioinformatics resources. *Nat Protoc* 2009; **4**: 44-57

6 Huang DW, Sherman BT, Lempicki RA. Bioinformatics enrichment tools: paths toward the comprehensive functional analysis of large gene lists. *Nucleic Acids Res* 2009; **37**: 1-13
